# Analysis of gene evolution and metabolic pathways using the Candida Gene Order Browser

**DOI:** 10.1186/1471-2164-11-290

**Published:** 2010-05-10

**Authors:** David A Fitzpatrick, Peadar O'Gaora, Kevin P Byrne, Geraldine Butler

**Affiliations:** 1 UCD School of Biomolecular and Biomedical Science, Conway Institute, University College Dublin, Belfield, Dublin 4, Ireland; 2 Department of Biology, The National University of Ireland, Maynooth, County Kildare, Ireland; 3 UCD School of Medicine and Medical Science, Conway Institute, University College Dublin, Belfield, Dublin 4, Ireland; 4 Smurfit Institute of Genetics, University of Dublin, Trinity College Dublin, Dublin 2, Ireland

## Abstract

**Background:**

*Candida *species are the most common cause of opportunistic fungal infection worldwide. Recent sequencing efforts have provided a wealth of *Candida *genomic data. We have developed the *Candida *Gene Order Browser (CGOB), an online tool that aids comparative syntenic analyses of *Candida *species. CGOB incorporates all available *Candida *clade genome sequences including two *Candida albicans *isolates (SC5314 and WO-1) and 8 closely related species (*Candida dubliniensis*, *Candida tropicalis*, *Candida parapsilosis*, *Lodderomyces elongisporus*, *Debaryomyces hansenii*, *Pichia stipitis*, *Candida guilliermondii *and *Candida lusitaniae*). *Saccharomyces cerevisiae *is also included as a reference genome.

**Results:**

CGOB assignments of homology were manually curated based on sequence similarity and synteny. In total CGOB includes 65617 genes arranged into 13625 homology columns. We have also generated improved *Candida *gene sets by merging/removing partial genes in each genome. Interrogation of CGOB revealed that the majority of tandemly duplicated genes are under strong purifying selection in all *Candida *species. We identified clusters of adjacent genes involved in the same metabolic pathways (such as catabolism of biotin, galactose and N-acetyl glucosamine) and we showed that some clusters are species or lineage-specific. We also identified one example of intron gain in *C. albicans*.

**Conclusions:**

Our analysis provides an important resource that is now available for the *Candida *community. CGOB is available at http://cgob.ucd.ie.

## Background

Fungal infections are the fourth most common nosocomial bloodstream infection in the United States. *Candida *species account for approximately 10% of all bloodstream infections [1] and worldwide are the most common cause of opportunistic fungal infection [2]. Due to their increasing clinical importance, recent sequencing projects have determined the complete sequence of ten *Candida *genomes, including common pathogenic species and species rarely, if ever, associated with disease [3-7].

**Figure 1:**
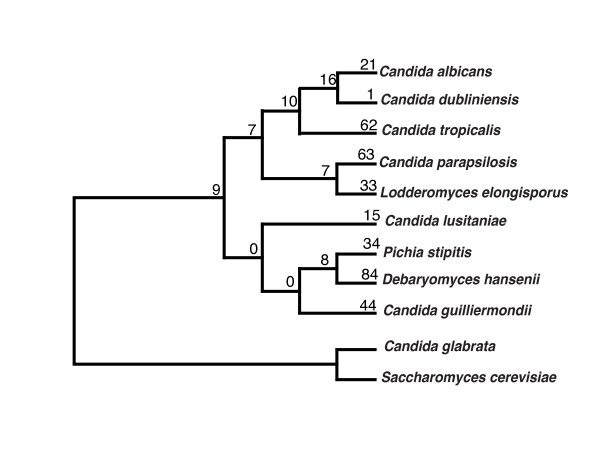
**Phylogenetic supertree of *Candida *species represented in CGOB**. *Candida glabrata *and *Saccharomyces cerevisiae *have been selected as outgroups. Numbers on branches represent tandem duplications gained along each lineage.

The term *Candida *was originally assigned to imperfect yeast species, with no known sexual cycle. This term now covers a variety of species of diverse origins (both sexual and asexual), and provides little information regarding evolutionary relationships. For example, *Candida glabrata *is more closely related to *Saccharomyces cerevisiae *than it is to *Candida albicans. Debaryomyces hansenii *and *Pichia stipitis *are close relatives of *Candida *species [8]. Some species, such as *C. lusitaniae*, were assigned two names, one (*Candida lusitaniae*) referring to the asexual (anamorph) form, and one (*Clavispora lusitaniae*) to the sexual (teleomorph) form. Similarly, *Candida guilliermondii *is also known as *Pichia guilliermondii*, and *Candida famata *as *D. hansenii*. These species share a relatively recent common ancestor (Figure 1), and in all cases the codon CUG is translated as serine rather than leucine [9]. For brevity, we refer to the above as *Candida *species that belong to the CTG clade [5,8,10].

Previous comparative analysis of eight *Candida *genomes led to the identification of gene families that are highly represented in strongly pathogenic species (such as *C. albicans, C. tropicalis, C. parapsilosis*), compared to weak pathogens such as *C. lusitaniae *and *C. guilliermondii*, and very rare or non-pathogenic species such as *D. hansenii *[5]. These include three cell wall families; the ALS-like adhesins, which in *C. albicans *have been associated with virulence, biofilm development and acquisition of iron from the host [11-13]; the Pga30-like family [14], and the Hyr/Iff family [15]. Many families are highly enriched for gene duplications [5].

There are currently two major browsers that display *Candida *genomes, CandidaDB, which contains information for several *Candida *genomes, and the Candida Genome Database, which predominantly describes *C. albicans *[16,17]. The major disadvantage of these browsers is that it is difficult to compare genomes to each other. They also display a to-scale representation of a chromosomal region, which is unsuitable for analysis of gene order and evolution. To overcome these problems we developed the Candida Gene Order Browser (CGOB [18]).

CGOB incorporates all available genome sequences from *Candida *species, including two isolates of *C. albicans *(SC5314 and WO-1), its close relative and minor pathogen *C. dubliniensis*, the major pathogens *C. tropicalis *and *C. parapsilosis*, the minor pathogens *L. elongisporus*, *C. lusitaniae *and *C. guilliermondii*, the marine yeast *D. hansenii*, and *P. stipitis*, a xylose-digesting yeast that is associated with beetles found in wood [3-7]. CGOB is based on the engine developed for the Yeast Gene Order Browser (YGOB) [19,20], which has been applied to the analysis of genome duplication in the *Saccharomyces *group. To construct CGOB, all assignments of homology were manually curated, based on sequence similarity and gene order (synteny). Partial genes in each genome were identified and removed, leading to the generation of improved gene sets. CGOB was then used to analyze gene duplication, intron localization and clustering of genes involved in metabolic pathways. We found that the majority of tandemly duplicated genes are under strong purifying selection and that there are both conserved and species-specific clusters of metabolically related genes in *Candida*. CGOB is available at http://cgob.ucd.ie.

## Results and discussion

### CGOB structure and *Candida *genome editing

**Figure 2:**
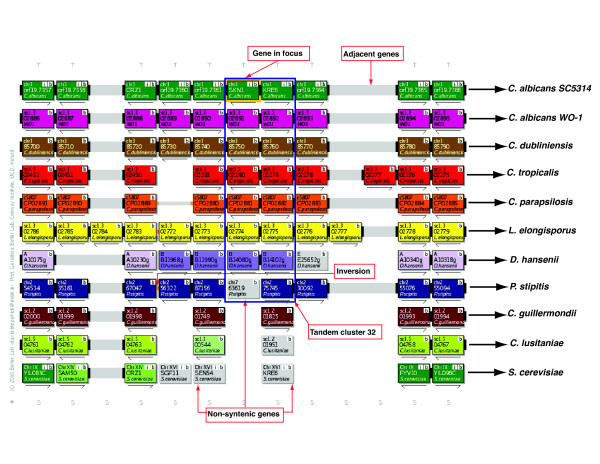
**Candida Gene Order Browser (CGOB) screenshot for tandem cluster 32**. Each box represents a gene and each color a chromosome (horizontal tracks). The gene in focus is highlighted with an orange border. Gene identifiers in the center of each box relate to annotations from the relevant sequencing centers. The "b" button performs a BLASTP search against CGOB's sequence database. The "S" button displays all the protein sequences in a vertical pillar. The "+" button outputs CGOB pillar data in a tabulated format. The "T" button reconstructs phylogenies on the fly. The "i" button retrieves functional data for *C. albicans *SC5314 and *S. cerevisiae via *the *Candida *Genome Database and the *Saccharomyces *genome database respectively. Non-syntenic genes are colored in grey. Genes lying in close proximity are joined by connectors: a solid bar for adjacent genes, two narrow bars connect genes up to 5 genes apart (not shown) and one narrow bar connects genes up to 20 genes apart (not shown). Inversions are denoted by orange connectors.

Version 1 of CGOB includes ten *Candida *genomes obtained from a variety of sequencing centers (Table 1), together with the genome from *S. cerevisiae*. CGOB's visual display consists of horizontal tracks representing chromosomal segments and pillars (Figure 2). Pillars are the core data structures used to store list of homologies across all species represented in the gene order browser [20]. Pillars contain vacant slots when homologous genes cannot be found in a particular genome. Genes were initially added to pillars based on automated assignments derived from best bidirectional BLASTP searches. The CGOB pillar dataset was manually refined by examining regions of dubious synteny and singleton genes. A combination of BLASTP scores, synteny and phylogenetic data were used to confirm assignments to pillars.

**Table 1:** *Candida *species displayed in CGOB.

Species	Citation	Genes	Partial ORFs	Refined Gene set	Singletons
*C. albicans *SC5314	[6]	6,185	0	6,185	43
*C. albicans *WO-1	[5]	6,197	91	6,148	99
*C. dubliniensis *CD36	[7]	5,924	0	5,924	200
*C. tropicalis *MYA-3404	[5]	6,258	116	6,198	737
*C. parapsilosis *CDC 317	[5]	5,823	28	5,809	553
*L. elongisporus *NRLL YB-4239	[5]	5,802	173	5,710	596
*P. stipitis *CBS6054	[3]	5,838	12	5,832	470
*D. hansenii *CBS767	[4]	6,317	12	6,311	981
*C. guilliermondii *ATCC6260	[5]	5,920	142	5,844	666
*C. lusitaniae *ATCC 42720	[5]	5,941	135	5,869	881

Similar to YGOB, CGOB allows the user to focus the screen display on a gene of interest and to view phylogenetic trees, sequences and BLASTP results (Figure 2). Hyperlinks to functional data can also be accessed for *C. albicans *SC5314 and *S. cerevisiae via *the *Candida *Genome Database [17] and the *Saccharomyces *Genome Database [21] respectively.

During manual editing of CGOB we observed that the original genome annotations contained a substantial number of apparently partial open reading frames, which we merged into full-length gene models. For example, *LELG_01495 *and *LELG_1496 *in *L. elongisporus *are both similar to parts of *C. albicans orf19.6045 *(*PSD1*). Closer inspection showed that *LELG_1496 *aligned with the N-terminus of *orf19.6045 *whereas *LELG_1496 *matches the C-terminus (Additional file 1). In cases like this we deleted the partial open reading frames from CGOB's homology pillars and inserted a new "merged" gene model. Overall, we identified 709 ORFs in 8 genomes that were subsequently merged into 335 full-length genes (Additional file 2). The *L. elongisporus *genome contained the highest number of partial ORFs (173 in total), while the highly curated genomes of *C. albicans *SC5314 and *C. dubliniensis *contain none (Table 1). The corrected gene sets are available for download from CGOB [18].

### Tandem Gene Duplications

Tandem gene duplication is one mechanism by which species acquire new genes, and by extrapolation, new functions. We therefore used both similarity and synteny measurements in CGOB to identify gene duplications in all *Candida *genomes. We identified and numbered all tandem clusters in each genome (Additional file 3). For some rapidly evolving genes, sequence similarity is not high enough to identify family members. For example, our initial BLAST based approach suggested that *orf19.2508 *(*PRM9*) and *orf19.2509 *in *C. albicans *are tandem duplicates (cluster 30, Additional file 3). Their orthologs in *C. dubliniensis*, *C. tropicalis*, *C. parapsilosis *and *L. elongisporus *are found adjacent to one another. However, these genes were not initially identified as tandem duplicates in the other species because they do not have a BLASTP *E*-value below our initial cut-off (See Methods). Therefore, slower evolving tandem duplicates in one *Candida *species (*C. albicans *SC5314 in this example) can be used to locate rapidly evolving tandems (or tandems with low sequence complexity) in other *Candida *genomes.

Tandem duplicates that subsequently underwent chromosomal rearrangement are difficult to identify. However, the ancestral arrangement can be inferred from an analysis of homologous genes in CGOB. For example, in *C. albicans *SC5314, the duplicate genes *orf19.7362 *(*SKN1*) and *orf19.7363 *(*KRE6*) are located beside one another on chromosome 3 (Figure 2, Additional file 3 (cluster 32)). Their orthologs in *C. albicans *WO-1, *C. dubliniensis*, *C. tropicalis*, *C. parapsilosis*, *L. elongisporus *and *D. hansenii *are also adjacent (Figure 2). However in *P. stipitis*, *SKN1 *(*PICST_63619*) is located on chromosome 7 while *KRE6 *(*PICST_75745*) is located on chromosome 2 (Figure 2). The most parsimonious explanation is that a duplication of *SKN1 *or *KRE6 *occurred in an ancestor of all the *Candida *species, and this has been conserved in most. However, relocation of *SKN1 *has occurred exclusively in *P. stipitis *(Figure 2).

In total 901 tandem clusters were identified across all the *Candida *genomes (Table 2, Additional file 3). *C. lusitaniae *has the smallest number of tandem clusters (44) whereas *C. parapsilosis *has the highest number (139). This is noticeably high, as the closest relative of *C. parapsilosis*, *L. elongisporus *(Figure 1), contains only 93 tandem clusters (Table 2). The average number of genes per tandem cluster in all *Candida *species ranges is slightly greater than 2 (Table 2).

**Table 2:** The total number of tandem duplicates found in each *Candida *species displayed in CGOB.

Species	Tandem Clusters	Species specific clusters	# of genes in clusters	Average # of genes per cluster	Tandem Duplicates
*C. albicans*	106	21	230	2.17	124 (1.99%)
*C. dubliniensis*	83	1	179	2.16	96 (1.62%)
*C. tropicalis*	125	62	284	2.27	159 (2.56%)
*C. parapsilosis*	139	63	328	2.36	189 (3.25%)
*L. elongisporus*	93	33	206	2.20	114 (1.99%)
*D. hansenii*	132	84	294	2.23	162 (2.56%)
*P. stipitis*	96	40	204	2.12	108 (1.85%)
*C. guilliermondii*	83	44	181	2.18	98 (1.67%)
*C. lusitaniae*	44	15	97	2.20	53 (0.90%)

Percentages in parenthesis refer to the total percentage of the genome that has arisen through tandem duplication.

We used CGOB to map species and lineage-specific tandem duplications (Figure 1). For example, since they last shared a common ancestor, *C. albicans *has undergone at least 21 species-specific tandem duplications gaining 24 paralogs, while its close relative *C. dubliniensis *has undergone a single tandem duplication (*Cd36_11890*, *Cd36_11900) *gaining 1 additional gene (Additional file 3, cluster 463). Similarly, *C. parapsilosis *has undergone 63 species-specific tandem duplications gaining 78 paralogs since diverging from its closest relative, *L. elongisporus*, which has undergone 33 tandem duplication gaining 41 paralogs in the same time (Additional file 3).

Cluster 22 (Additional file 3) illustrates an ancient duplication, resulting in a family of peroxisomal acyl-CoA thioesterases that are present in 3-5 tandem copies in all the *Candida *species. The cluster is particularly large in the branch containing *C. albicans, C. dubliniensis, C. tropicalis, C. parapsilosis *and *L. elongisporus*, where 5 family members are immediately adjacent to each other. The single homolog in *S. cerevisiae *is likely to be involved in fatty acid oxidation [22]. There is significant up-regulation of fatty acid β-oxidation when *C. albicans *cells are engulfed by macrophages [23], although this pathway does not appear to be essential for virulence [24]. Many of the other tandem duplication clusters include members of larger gene families, such as lipases (cluster 10, Additional file 3), glucose transporters (cluster 15,61, Additional file 3) and ferric reductases (cluster 57, Additional file 3). Some clusters are lineage specific, such as the triplication of the pirin-domain genes *PRN2, PRN3 *and *PRN4 *(cluster 6, Additional file 3) in *C. albicans*, *C. dubliniensis *and *C. tropicalis*. The function of these genes in unknown, but they are likely to localize to the nucleus. There is an amplification of the *FRP6 *family in *D. hansenii *and *P. stipitis*; the *S. cerevisiae *orthologs are required for export of ammonia [25] (cluster 497, Additional file 3). Other clusters are species-specific, such as the five adjacent 2' hydroxyisoflavone reductases (CIP1) described by Jeffries and Van Vleet [26], which our analysis confirms is unique to *P. stipitis *(cluster 228). Most of the other species have a single copy, except for *C. parapsilosis*, which has two. *L. elongisporus *contains 5 tandem repeats of a large family with up to 13 members in this species, which is absent from all the other *Candida *genomes (cluster 280, Additional file 3). The function of this family is unclear but all members contain a Phosphatidylinositol Phosphate Kinase (PIPKc) domain.

We also determined whether tandem duplicates in individual *Candida *species are undergoing positive selection. Recent genome wide studies have shown that positive selection after tandem duplication can give rise to novel gene functions [27], that may help pathogens evade the human immune response [28]. At the DNA level, positive selection may be detected by comparing the rate of amino acid altering (nonsynonymous) nucleotide substitutions with the rate of synonymous substitution (d_N_/d_S_). A d_N_/d_S _ratio > 1 is indicative of positive selection. The average d_N_/d_S _ratio for all tandem clusters was found to be 0.27 (not shown). Of the 901 *Candida *clusters examined, only 12 displayed a d_N_/d_S _ratio > 1 (Additional file 3). Five of these are species-specific and have no homologs in any other *Candida *species (or any species in GenBank). The remaining 7 clusters under the influence of positive selection do not share homology with gene families (cell wall, hyphal, pseudohyphal, filamentous growth and biofilm functions) normally associated with pathogenicity in *Candida *[5]. In *P. stipitis*, one cluster encodes putative ubiquitin protein ligases, one encodes zinc finger-containing proteins, and one encodes potential siderophore transporters (Additional file 3). In *D. hansenii*, one cluster encodes orthologs of *TFS1*, whose expression is induced during filamentation in *C. albicans *[29]. Overall our results suggest that the majority of *Candida *tandem duplicates are under the influence of strong purifying selection, presumably to conserve gene function.

We have extended an earlier analysis of duplicate genes in *Candida *genomes, which considered only members of multigene families [5]. We also identified some clusters by manual inspection. For example, we first identified 85 tandem clusters in *C. albicans *SC5314 using a simple BLAST approach, and this was increased to 106 using synteny information, whereas only 24 were reported in Butler et al [5]. In some species (such as *L.elongisporus *and *C. guilliermondii*) we identified a smaller number of clusters than Butler et al [5], partly because we removed partial ORFs from the gene sets.

### The *Candida *Paranome

Using our reannotated *Candida *genomes we determined the number of multigene families (the paranome [30]) for each *Candida *species. *C. tropicalis *has the highest number (557), whereas *C. lusitaniae *(390) has the lowest (Table 3). In contrast, *C. parapsilosis *has the lowest number (4377) of genes that do not belong to families, whereas *C. lusitaniae *(4871) has the highest (Table 3). The average number of genes per multigene family is approximately 3 for all species, although all *Candida *species have larger gene families (Table 3).

**Table 3:** The *Candida *paranome.

	Unique genes	Multigene Families	Average # genes per family	Gene families containing			
				
				2 members	3 members	4 members	>5 members
*C. albicans *SC5314	4662 (76.3%)	484	2.99	10.0%	3.9%	2.7%	7.1%
*C. albicans *W01	4556 (76.8%)	467	2.95	10.3%	3.4%	2.7%	6.8%
*C. dubliniensis*	4449 (75.1%)	513	2.87	11.4%	3.8%	3.0%	6.7%
*C. tropicalis*	4603 (73.6%)	557	2.97	11.8%	3.7%	2.6%	8.3%
*C. parapsilosis*	4377 (75.2%)	472	3.06	10.5%	3.5%	2.4%	8.4%
*L. elongisporus*	4616 (79.6%)	413	2.86	9.3%	3.6%	2.2%	5.3%
*D. hansenii*	4808 (76.2%)	519	2.89	11.1%	3.7%	2.3%	6.7%
*P. stipitis*	4335 (74.3%)	497	3.02	10.5%	4.6%	2.8%	7.8%
*C. guillermondii*	4558 (77.0%)	473	2.87	10.5%	4.5%	1.6%	6.4%
*C. lusitaniae*	4871 (82.0%)	390	2.74	9.3%	3.0%	1.6%	4.1%

The number of single- and multi- gene families in each *Candida *species displayed in CGOB.

The largest gene family shared by all species contains a transporter (*DIP5*) annotated as a putative dicarboxylic amino acid permease in CGD. This family was previously suggested as a potential antifungal target, as there are no homologs in humans [31]. All *Candida *species have at least 20 members of this family (not shown). The *MEP *family, encoding three ammonium permeases in *C. albicans *SC5314, has also suggested as an antifungal drug target [31]. Three *MEP *genes are present in all *Candida *species except for *L. elongisporus*, which is missing the ortholog of one (*orf19.4446*, not shown). Drugs directed against these families should therefore be of broad specificity and target all *Candida *species, and are likely to have no undesired interactions with the human patient.

Approximately 20-25% of all *Candida *genes in CGOB belong to a multigene family (Table 3), similar to what has previously been reported for *C. albicans *SC5314 [31]. This figure is lower than what has been observed for *S. cerevisiae *(~30%), which is unsurprising as *S. cerevisiae *has undergone a whole genome duplication [32] while *Candida *species have not [33].

### Intron loss in *Candida genes*

Yeast genomes from the Saccharomycotina are known to be intron poor; introns are found in fewer than 5% of genes from most species [34]. The exact mechanisms of intron loss are not fully elucidated, but it is likely to occur via recombination of a chromosomal copy with a reverse transcript [35,36]. Introns have been predicted with some accuracy in only three of the sequenced *Candida *genomes - *C. albicans*, *C. dubliniensis *and *D. hansenii*. We therefore restricted our analysis to these species. Where we observed differences in intron locations in tandem duplicates in any one species, the corresponding genomic sequence of the other two was manually inspected to confirm intron presence or absence.

*C. albicans *SC5314 has at least 381 genes containing 415 introns [37]. Of these genes, 79 (~21%) belong to a multigene family, and five are located in tandem clusters.

**Figure 3:**
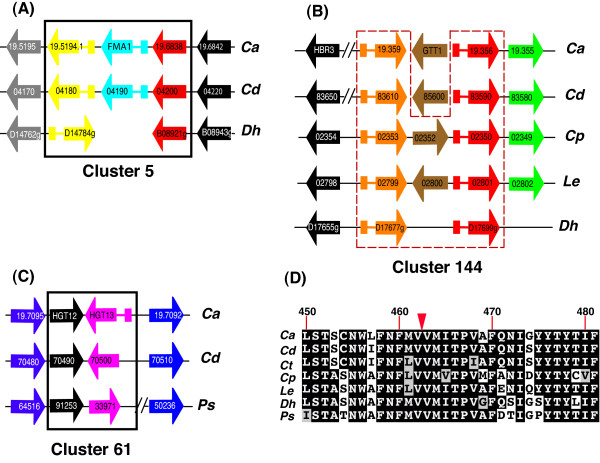
**Gene order around tandem clusters 5, 144 and 61 (A, B, C respectively)**. The diagram is re-drawn from CGOB with some species omitted for clarity. Homologs are organized in pillars. Intron containing genes are indicated with a tail. A) Cluster 5. *D. hansenii *does not contain an ortholog of FMA1. B) Cluster 144 is represented by a broken red line. GTT1 (*orf19.6998*) and *Cd36_85600 *from *C. albicans *and *C. dubliniensis *are not part of this cluster, and are located elsewhere in the genome. Diagonal lines indicate a gap of 2 and 4 genes in *C. albicans *and *C. dubliniensis *respectively. C) Cluster 61. Diagonal lines indicate a gap of 23 genes in *P. stipitis*. D) Partial alignment around the *C. albicans *HGT13 intron. The position of the intron in *C. albicans *is indicated with an inverted triangle.

Cluster 5 contains three paralogs in *C. albicans *(*orf19.5194.1*, *orf19.6837 *(*FMA1*), and *orf19.6838*) (Additional file 3 and Figure 3A). The first two of these genes contain introns, as do their orthologs in *C. dubliniensis*. There is a single homolog in *D. hansenii*, which has undergone an inversion relative to *C. albicans*, but still contains an intron. However, the third gene (*orf19.6838*) does not contain an intron in any of the species (Figure 3A). The most likely hypotheses are that either the progenitor copy contained an intron and was duplicated twice, followed by intron loss in one copy, or that the progenitor was first duplicated to generate a second intron-containing copy, and then duplicated again in an RNA-mediated event.

Cluster 144 (Additional file 3) contains members of a family of glutathione-S-transferases that are present in two adjacent copies in some species and three in others (Figure 3B). Two genes in *C. albicans*, C. *dubliniensis *and *D. hansenii *have introns, suggesting that they arose through tandem duplication (Figure 3B). However, a third member of the family (*GTT1*) which lies within the cluster in *C. parapsilosis*, *L. elongisporus *and *C. guilliermondii *does not contain any introns in *C. albicans *or *C. dubliniensis *(there is no ortholog in *D. hansenii*). *GTT1 *may therefore have arisen via an RNA intermediate in an ancestral *Candida *species.

We also found evidence for a species-specific intron gain in a tandem duplicate. Cluster 61 (Additional file 3) contains two adjacent genes (*HGT12 *and *HGT13*) in *C. albicans*, *C. dubliniensis *and *P. stipitis *that belong to a large family of sugar transporters that have at least 20 members in *Candida *species [38] (Figure 3C). The *HGT13 *homolog is not adjacent to *HGT12 *in the other species (not shown). In *C. albicans*, *HGT13 *contains an intron, whereas its paralog *HGT12 *does not. The *HGT13 *intron lies within the coding sequence, which makes it easy to identify (Figure 3D). Interestingly, although this intron is present in *HGT13 *from both *C. albicans *isolates, it is absent from all of the homologs in the other species, whether syntenic or not (Figure 3C). Intron gain is very rare [36,39], but it appears more likely that *HGT13 *in *C. albicans *gained an intron, rather than the intron was independently lost from all the other species. At least one other member of the HGT family (*HGT9*) also contains introns in *C. albicans*, but these appear to be conserved in *C. dubliniensis *only.

Other gene families that are not tandemly arranged are also likely to have arisen through both DNA-based gene duplication and via an RNA intermediate, possibly including retrotransposition. For example in *C. albicans*, the glycosylphosphatidylinositol-linked cell wall gene *ECM33 *[40] has two paralogs *orf19.4955 *and *orf19.4255 *(*ECM331*) that are not adjacent to each other (not shown). *ECM33 *and *orf19.4955 *both contain an intron, as do their orthologs in *C. dubliniensis *and *D. hansenii. ECM331 *does not contain an intron in any of the three species. This suggests that *ECM33 *and *orf19.4955 *may have arisen through duplication, and *ECM331 *is the result of reverse transcription of one of the intron-containing paralogs, in an ancestor of the three species.

### Clustering of adjacent genes in metabolic pathways

In bacteria the primary method of controlling gene expression is the organization of genes into operons, which are transcribed into a single mRNA. Bacterial operons often contain genes from the same metabolic pathway. Operons are not usually found in eukaryotes, with the notable exception of nematodes [41-43]. However, there is evidence for clustering of genes at the same genomic location belonging to the same metabolic pathways in fungi. For example, genes involved in secondary metabolism are clustered in the genomes of filamentous ascomycetes [44], and many of the genes involved in metabolism of allantoin and galactose are clustered in the genome of *S. cerevisiae *and related species [45,46]. Many functionally-related genes are co-expressed, even when they do not share sequence similarity [26]. Lee and Sonnhammer [47] found that there is significant tendency for genes from the same metabolic pathway to cluster in the genomes of fungi, and in other organisms. However, their definition of proximity was very large, and included genes that were separated by up to 400 other genes. Our analysis had a more focused approach, as we searched for evidence of genes involved in the same metabolic pathway lying up to 10 genes apart in *Candida *species.

Currently there are 155 metabolic pathways that have been manually curated by the *Candida *Genome Database. However, 23 of these contain only one gene, and a further 33 are redundant. For example, the list of genes involved in the acrylonitrile and aldoxime degradation pathways are identical. Similarly the tyrosol, tryptophan, phenylalanine and chorismate biosynthesis are all subsets of the superpathway of phenylalanine, tyrosine and tryptophan biosynthesis. There are 99 unique pathways, containing 659 genes. There are 511 unique genes in total, representing 8.2% of the *C. albicans *SC5314 gene set.

CGOB was interrogated for evidence of clustering of genes (i.e. lying within 10 genes of one another on the same chromosome) in the 99 nonredundant pathways. We identified 21 pathways that display evidence of gene clustering in at least one *Candida *species (Table 4 and Additional file 4). Some metabolic pathway clusters result from tandem duplication; for example, *AOX1 *and *AOX2 *(encoding cyanide insensitive enzymes required for an alternative pathway of aerobic respiration) in *C. albicans*, *C. dubliniensis*, *C. tropicalis *and *C. parapsilosis*, were also identified as tandem cluster 9 (Additional file 3). There is evidence of species-specific clusters of unrelated genes, such as lysine biosynthesis and glycine biosynthesis, which are clustered in one species only (*C. tropicalis *and *C. parapsilosis *respectively, Table 4, Additional file 4).

**Table 4:** CGD metabolic pathways that show evidence of gene clustering.

	#Genes	SC5314	WO1	Cdub	Ctro	Cpar	Lelo	Dhan	Psti	Pgui	Clus
Histidine, purine and pyrimidine biosynthesis	41	7	7	7	5	4	4	-	3	4	-
Aerobic respiration (cyanide sensitive)	14	3	3	3	3	2	3	3	3	2	2
Aerobic respiration (cyanide insensitive)	8	2	2	2	2	2	-	-	-	-	-
2-keto glutarate dehydrogenase complex	3	2	2	2	2	-	-	-	2	-	-
N-acetylglucosamine degradation	3	3	3	3	3	3	3	3	3	3	3
Methylglyoxal pathway	12	2	2	2	2	2	2	-	-	-	-
Sphingolipid metabolism	8	-	-	-	-	-	-	-	-	2	-
Ergosterol biosynthesis	21	-	-	-	-	-	-	2	-	2	2
Biotin biosynthesis	4	4	4	-	3	-	-	2	-	-	-
NAD salvage pathway	4	-	-	-	2	-	-	-	-	-	-
Tetrapyrrole biosynthesis	4	2	2	2	2	-	-	-	2	-	-
Pantothenate and coA biosynthesis	11	2	2	2	2	2	2	2	2	2	2
Starch degradation	7	-	-	-	-	-	-	-	-	-	2
Lipid-linked oligosaccharide biosynthesis	8	-	-	-	2	2	3	2	3	2	-
Arginine degradation (arginase pathway)	5	2	2	2	-	-	-	2	2	2	2
Lysine biosynthesis	6	-	-	-	2	-	-	-	-	-	-
Superpathway of glycine biosynthesis	5	-	-	-	-	2	-	-	-	-	-
Biosynthesis of phe/tyr/trp	13	-	-	-	-	-	2	-	-	-	-
Superpathway of glycine biosynthesis	5	-	-	-	-	5	-	-	-	-	-
Acrylonitrile degradation	4	-	-	-	2^(2T)^	2^(2T)^	2^(2T)^	-	2^(2T)^	-	-
Galactose degradation	5	4^(2D)^	4^(2D)^	4^(2D)^	4^(2D)^	4^(2D)^	4^(2D)^	4^(2D)^	4^(2D)^	4^(2D)^	4^(2D)^
tRNA charging pathway	35	13^(6D)^	13^(6D)^	13^(6D)^	10^(2D)^	7^(4D)^	9^(6D)^	9^(4D)^	13^(6D)^	9^(4D)^	5^(4D)^
Fatty acid oxidation pathway	14	4^(4D)^	4^(4D)^	2^(2D)^	4^(4D)^	4^(4D)^	-	2^(2D)^	2^(2D)^	2^(2T)^	2^(2T)^
Glutathione-glutaredoxin redox reactions	9	5^(3D, 3D)^	5^(2D,2T)^*	6^(3D,3T)^	3^(3T)^	4^(4T)^	3^(3T)^	2^(2T)^	2^(2T)^	3^(3T)^	2^(2D)^
Isoleucine & phenylalanine degradation	12	2^(2T)^	2^(2T)^	-	-	-	-	-	-	-	-
Removal of superoxide radicals	7	2^(2T)^	2^(2T)^	2^(2T)^	-	-	-	-	-	-	-

Superscript numerals in parenthesis refer to the numbers of genes that are either paralogs ^D ^or tandem duplicates ^T^.

A high proportion (48%) of the clusters identified contain only two genes and may not be biologically significant, as they appear at a high frequency in randomized data (see Methods). However, the metabolic clusters discussed here are highly significant, particularly for the three pathways discussed below.

### (i) The biotin biosynthesis pathway

Biotin or vitamin H acts as a cofactor for a set of enzymes that catalyze carboxylation, decarboxylation, and transcarboxylation reactions in a number of crucial metabolic processes [48]. Most multicellular eukaryotes (except for plants) are biotin auxotrophs, whereas many bacterial species and some fungi (including *Aspergillus *and *Saccharomyces *species) are biotin prototrophs [49,50].

In *S. cerevisiae *6 genes are involved in the production of biotin (*BIO1-6*). These are located in 2 clusters (*BIO1*/*BIO6 *and *BIO3*/*BIO4*/*BIO5*, with *BIO2 *at a different location) [49]. Hall and Dietrich [49] showed that the original eukaryotic biotin pathway was lost in the last common ancestor of *Candida *and *Saccharomyces *species, but it has been rebuilt through horizontal gene transfer from bacterial species via transfers of *BIO3 *from δ-proteobacteria and *BIO4 *from α-proteobacteria, followed by gene duplication and neofunctionalization.

**Figure 4:**
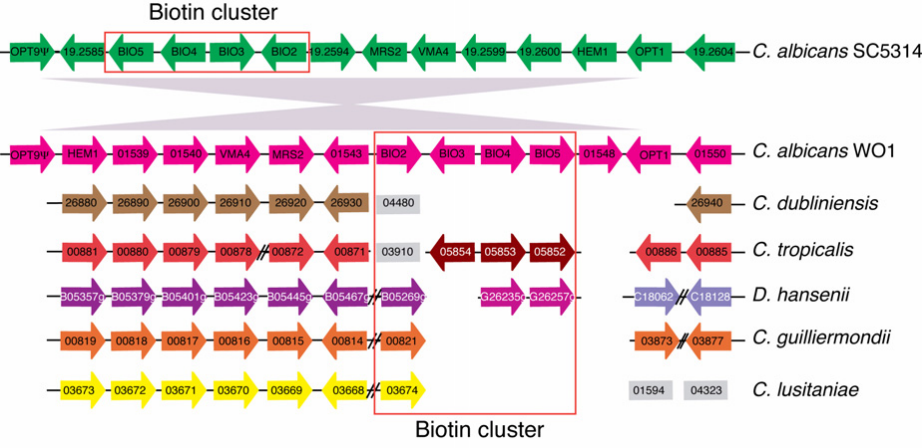
**Gene order around the biotin cluster**. The diagram is re-drawn from CGOB with some genes and species omitted for clarity. Blocks of color represent chromosomes. Homologs are organized in pillars. Changes in color indicate breaks in synteny. The grey triangles indicate an inversion between the *C. albicans *SC5314 and WO-1 isolates. Diagonal lines indicate local inversions. Genes shown in grey boxes are not adjacent to any other gene shown.

We identified a biotin cluster of four genes (orthologs of *S. cerevisiae BIO2*, *BIO3*, *BIO4 *and *BIO5*) in both *C. albicans *strains (Figure 4). There is however an inversion of the surrounding region between SC5314 and WO-1 (Figure 4); this appears to result from a rearrangement between two members of the oligopeptide transporter gene family, *OPT9 *(a pseudogene) and *OPT1*.

The cluster in *C. albicans *is larger than the equivalent region in *S. cerevisiae *as it includes *BIO2. BIO2 *orthologs are in the same chromosomal region in *C. albicans*, *D. hansenii*, *C. lusitaniae *and *C. guilliermondii *(Figure 4). *BIO3, BIO4 *and *BIO5 *are also adjacent to each other in *C. tropicalis*, and they appear to have been recruited to the *BIO2 *region in *C. albicans*. Almost the entire cluster, together with an adjacent *OPT *gene, is missing from *C. dubliniensis*. Only *BIO2 *remains, and this is located elsewhere in the genome. The absence of the biotin cluster in *C. dubliniensis *has previously been reported, and it was suggested that its presence in *C. albicans *may contribute to increased prevalence and virulence [51]. The entire set of *BIO *genes is also absent from *C. parapsilosis *and *L. elongisporus*, and was probably lost in their last common ancestor (not shown). There is some conservation of synteny of the surrounding genes (not shown), suggesting the genes were lost together, as a cluster. Unlike *S. cerevisiae*, the biotin clusters in *C. albicans *and *C. tropicalis *are not sub-telomeric.

The remaining *Candida *species contain some genes involved in biotin synthesis. *BIO2 *is present in almost all species, suggesting it may play a role independent of biotin synthesis. *BIO4 *and *BIO5 *are clustered in *D. hansenii *with *BIO2 *elsewhere in the genome, whereas *BIO2, BIO3 *and *BIO4 *are present in *P. stipitis*, but are not clustered (not shown). It is not clear why some components of the pathway are retained in some species. However, it may enable them to make biotin from some intermediates, as was described for *S. cerevisiae *[52]. It is generally assumed however that clustering of genes in biosynthetic pathways is the result of selection against toxic intermediates produced by incomplete pathways [49]. It is likely that the ancestral *Candida *species was able to synthesize biotin, but there has been substantial gene loss in many species.

In *S. cerevisiae BIO6 *is believed to have arisen through gene duplication of *BIO3 *followed by subfunctionalization [49]. We cannot locate an ortholog of *BIO6 *in any *Candida *species. Similarly we cannot locate any *Candida *ortholog of *BIO1 *(pimeloyl-CoA synthetase), the first enzyme involved in synthesizing biotin from pimelic acid. In *S. cerevisiae *S288C, *BIO1 *and *BIO6 *are pseudogenes [49], but there is no evidence of corresponding pseudogenes in any *Candida *species. It is therefore unlikely that the genes are present in other unsequenced isolates of the same species.

The CGD biotin pathway data suggests that *orf19.3567 *(*BIO32*) is involved in biotin synthesis. *BIO32 *has a top BLASTP hit to *BIO3 *in *S. cerevisiae*. However, *BIO3 *belongs to a multigene family that also contains *ARG8*, *CAR2 *and *UGA1*. To determine the origin of *BIO32 *we reconstructed a phylogenetic tree using the same sequences used by Hall and Dietrich [49], and included *ARG8*, *CAR2 *and *UGA1 *from *C. albicans *and *S. cerevisiae*. Our phylogeny places the *S. cerevisiae *and *C. albicans BIO3 *orthologs together with bacterial sequences, indicating that they originated from horizontal gene transfer as suggested by Hall and Dietrich [49]. *S. cerevisiae BIO6 *is also grouped in this clade, supporting the hypothesis that it is a duplicate of *BIO3*. However, *BIO32 *from *C. albicans *is grouped with *S. cerevisiae *and *C. albicans *orthologs of *ARG8*, *CAR2 *and *UGA1 *in a separate clade (not shown). *BIO32 *is therefore most likely a duplicate of one of these genes, and is more likely to be involved in arginine or glutamate metabolism than in biotin synthesis.

### (ii) The *N*-acetylglucosamine regulon

It has been proposed that the ability of pathogenic strains of *Candida *to utilize sugars such as glucosamine and *N*-acetylglucosamine (Nag) as alternative carbon sources are important virulence factors [53]. *C. albicans *mutants incapable of utilizing Nag are less virulent in a murine model of systemic candidiasis compared to wild type isolates [54]. The three genes involved in the conversion of Nag to fructose-6-phosphate encode hexokinase kinase (*HXK1/orf19.2154*), Nag-6-phosphate deaminase (*NAG1/orf19.2156*) and Nag-6-phosphate deacetylase (*DAC1/orf19.2157*). These act sequentially on Nag and are present in *C. albicans *in a cluster termed the Nag regulon [53].

**Figure 5:**
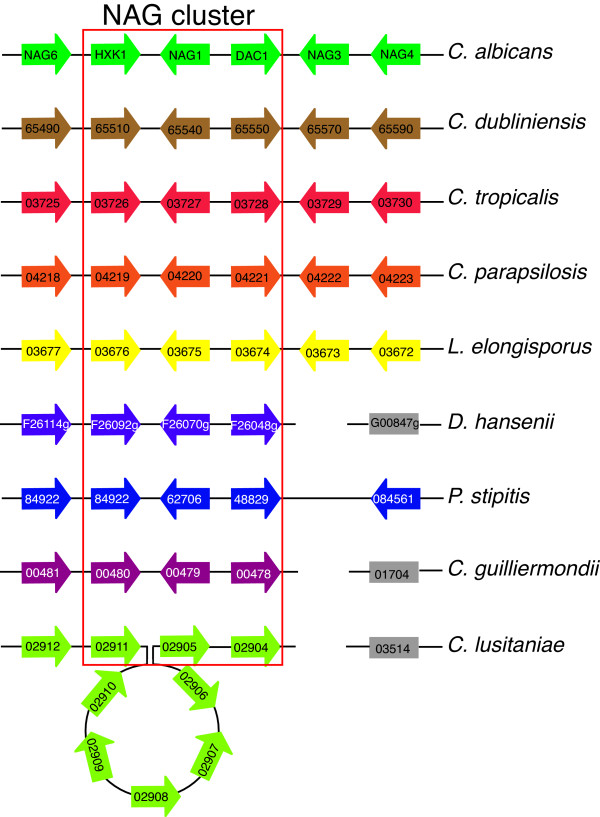
**Gene order around the *N*-acetylglucosamine (NAG) cluster**. The diagram is re-drawn from CGOB. Blocks of color represent chromosomes, homologs are organized in pillars and genes shown in grey boxes are not adjacent to any other gene shown. Synteny of NAG enzymes is observed in all species except for *C. lusitaniae*, which has 5 intervening genes displayed as an insertion loop.

Our clustering analysis shows that the Nag regulon is conserved in all *Candida *species, with the exception of *C. lusitaniae *(Figure 5, Additional file 4). In the latter species, there has been an insertion of 5 species-specific genes in the region between *HXK1 *and *NAG1*, resulting in a sequence of 19,931 basepairs (bp), whereas the intergenic region in the other species is less than 516 bp. Several of the inserted genes encode members of a family of cell wall genes, related to *Flo1 *from *S. cerevisiae*. The Nag cluster is sub-telomeric in many of the *Candida *species, and repeats of cell wall genes are commonly found near telomeres [55]. The conservation of the Nag regulon in pathogens like *C. albicans *and nonpathogens such as *P. stipitis *suggests that the ability to utilize Nag is not a virulence factor. *NAG3*, *NAG4 *and *NAG6*, which lie close to the NAG cluster in many *Candida *species (Figure 5), are not involved in the conversion of Nag, but are more likely to encode drug efflux pumps [56,57]. *NAG3 *is a tandem duplicate of *NAG4 *(Additional file 3, cluster 59), which occurred in the ancestor of *C. albicans, C. dubliniensis*, *C. tropicalis*, *C. parapsilosis *and *L. elongisporus *(Figure 1).

The phylogenomic distribution of the Nag regulon is intriguing. The cluster is found in the *Candida *species, but *NAG1 *and *DAC1 *are missing in the *Saccharomyces *lineage. Homologs are also absent from *Ashbya gossypii *and *Kluyveromyces waltii*, suggesting the cluster is missing from the entire Saccharomycetes lineage. However, the origin of the NAG cluster may be an ancient event. *DAC1 *and *NAG1 *are in close proximity (within two genes) in the *Aspergilli *and in *Neurospora crassa*, which belong to the Pezizomycotina, a sister clade to the Saccharomycotina. *DAC1 *and *NAG1 *also lie within 2 genes in the Basidiomycete, *Ustilago maydis*. If the cluster arose in an ancestor of the Ascomycota and the Basidiomycota, it is very ancient, and the genes have been subsequently lost from many species (including *Schizosaccharomyces*).

### (iii) The Leloir galactose utilization pathway

**Figure 6:**
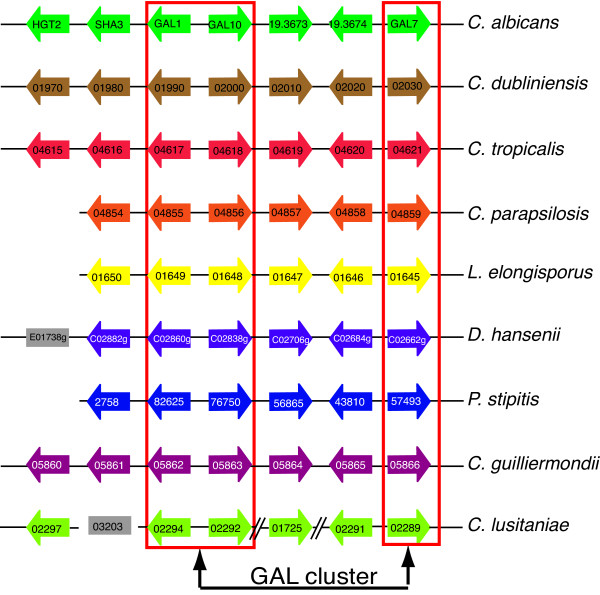
**Gene order around the Galactose (GAL) cluster**. The diagram is re-drawn from CGOB. Blocks of color represent chromosomes and homologs are organized in pillars. Diagonal lines indicate local inversions. Genes shown in grey boxes are not adjacent to any other gene shown. Dubious genes (*orf19.3671 *and *CLUG_02293*) from *C. albicans *and *C. lusitaniae *are not displayed.

Galactose is utilized by most organisms through its conversion to glucose-6-phosphate, which then enters glycolysis [58]. The GAL pathway is composed of both structural and regulator elements [59]. The galactose metabolism structural genes of *S. cerevisiae *and *C. albicans *are well conserved, whereas their regulatory components are distinct [59]. In *C. albicans *the structural genes (*GAL1*, *GAL10 *and *GAL7*) are arranged in a cluster close to a hexose transporter *HGT2 *[59]. This cluster, together with two additional uncharacterized genes which lie between *GAL10 *and *GAL7*, is conserved in *C. albicans*, *C. dubliniensis*, *C. parapsilosis *and *D. hansenii *[59]. We show that the GAL pathway cluster is conserved in all *Candida *species present in CGOB (Figure 6).

Both *C. albicans *strains and *C. lusitaniae *have a gene insertion between *GAL1 *and *GAL10 *(not shown). The *C. albicans *gene (*orf19.3671*) is designated "dubious " by CGD, and is a pseudogene in WO-1, with no significant similarity to any other gene known from any other organism. Similarly the *C. lusitaniae *gene (*CLUG_02293*) has no significant homologs in either GenBank or CGOB Blast databases. The intergenic regions between *GAL1 *and *GAL10 *are 490 and 1362 nucleotides in *C. lusitaniae *and *C. albicans*, similar to the intergenic regions in all the other *Candida *species. It is likely therefore that both *orf19.3671 *and *CLUG_02293 *are errors in annotation, rather than real genes and so are not shown in Figure 6.

Expression of the hexose transporter *HGT2 *is strongly induced by galactose in *C. albicans *[59]. An ortholog of *HGT2 *is also very close to *GAL1 *in *C. dubliniensis*, *C. tropicalis*, *C. guilliermondii *and *C. lusitaniae *(Figure 6). *HGT2 *belongs to a large gene family, and while multiple homologs were located in many species, there is no family member adjacent to the GAL cluster in *C. parapsilosis, L. elongisporus *and *P. stipitis*. A putative ortholog in *D. hansenii *was identified, although it resides on a different chromosome to the GAL genes (Figure 6). It is possible that even though the relative position of the hexose transporter is not conserved, co-expression with the GAL genes may be.

Interestingly, *orf19.3674*, which lies between *GAL10 *and *GAL7*, appears to be a paralog of *GAL10*, and is conserved in all the *Candida *species. This was also noted in the *P. stipitis *genome [3]. Gal10 is more than twice the size of orf19.3674 (675 *vs*. 320 amino acids), and contains two recognized protein domains, an NAD dependent epimerase/dehydratase domain and an aldose 1-epimerase domain. Only the first domain is present in *orf19.3674 *and its orthologs. Expression of this gene is not influenced by galactose in *C. albicans *[59]. *orf19.3674 *may therefore have undergone subfunctionalization after duplication. Alternatively, recombination between a *GAL10 *precursor and another gene may have led to gene with a novel function. An ortholog of the adjacent conserved gene (*orf19.3673*) encodes a subunit of the transport protein particle (TRAPP) of the cis-Golgi in *S. cerevisiae *[60] and is unlikely to be involved in galactose metabolism.

### KEGG Analysis

We also assigned genes in each *Candida *species to individual metabolic pathways using the Kyoto Encyclopedia of Genes and Genomes (KEGG) [61]. This approach permitted us to investigate species-specific metabolic pathways, as well as pathways that have been described only in *C. albicans *SC5314. Approximately 200 metabolic pathways were reconstructed for each *Candida *species (Table 5). However, ~30% of these were redundant (Table 5). For example, in *C. dubliniensis*, the inferred components of the pathways for peptidoglycan and alkaloid biosynthesis are completely contained in the alanine, aspartate and glutamate metabolism pathway (not shown). The number of gene assignments to the non-redundant pathways is approximately 2000 for each *Candida *species. On average close to 50% of these are represented in multiple pathways (Table 5). Therefore between 16-19% of genes from each *Candida *species have been successfully assigned to a unique KEGG metabolic pathway (Table 5), equating to 17.8% of all *Candida *genes represented in CGOB.

**Table 5:** KEGG metabolic pathways that show evidence of gene clustering in *Candida *species.

Species	Pathways	Non-Redundant pathways	Genes in Pathways	Unique Genes in Pathways	Metabolic clusters
*C. albicans*	190	136	1870	991 (16.0%)	38
*C. dubliniensis*	196	139	1857	968 (16.3%)	39
*C. tropicalis*	201	139	1864	988 (15.9%)	35
*C. parapsilosis*	204	149	2026	1062 (18.3%)	34
*L. elongisporus*	202	142	1991	1048 (18.4%)	33
*D. hansenii*	209	152	2165	1148 (18.2%)	39
*P. stipitis*	205	151	2134	1114 (19.1%)	44
*C. guilliermondii*	210	158	2162	1126 (19.3%)	36
*C. lusitaniae*	207	146	2069	1091 (18.6%)	34

We interrogated each *Candida *species in CGOB for evidence of clustering in the non-redundant KEGG pathways (Table 5). In total we identified 62 pathways; (33-44 per species) that display some evidence of gene clustering (Table 5). There are 767 KEGG clusters (KCs) shared amongst all species, and of these 32 have arisen through tandem duplication (Additional file 5). Most of the identified KCs are small, containing two or three genes. A high proportion (75%) of these may not be biologically significant (Additional file 4), as they appear at a high frequency in randomized data (see Methods).

Overall the observed KEGG metabolic pathway clusters are generally distinct from those located using the CGD pathways (Additional file 4 and Additional file 5). There is a small degree of crossover, including the CGD tRNA charging pathway, which is analogous to KEGG's aminoacyl-tRNA biosynthesis pathway, the CGD aerobic respiration pathway which is equivalent to oxidative phosphorylation in KEGG, and the galactose metabolism and histidine metabolism pathways in both. Several pathways (such as clustering of histone protein genes (ko05322) and ribosomal protein genes (ko03010) have been described previously. It is likely that other clusters will be identified when the assignments to pathways improve. For example, Jeffries and Van Vleet [26] identified some small clusters of functionally-related genes in *P. stipitis *by visual inspection. Our approach found some of these, but not all.

## Conclusions

We describe here a unique tool for studying evolution and gene function in *Candida *species. During the development of CGOB we improved the existing annotations for several species, by identifying and removing partial open reading frames, and by manually assigning homology, based on sequence similarity and synteny. We also provide a detailed analysis of gene clusters in *Candida*, which will provide a basis for future investigation. We identified many of the clusters described in only one species [3,4,26,53,59]. However, we have also shown the benefits of a comparative approach; some clusters (such as NAG) although originally described in *C. albicans *only are present in all *Candida *species, whereas others (such as CIP) are unique to one (*P. stiptis*). Our analysis provides an important resource that is now available for the *Candida *community.

## Methods

### Genome Data

The complete *C. albicans *(SC5314) genome (Assembly 21 [31]) was obtained from the *Candida *genome database (CGD) [62]. Gene sets for *C. albicans *WO-1, *C. tropicalis*, *L. elongisporus*, *C. guilliermondii*, and *C. lusitaniae *[5] were obtained directly from the Broad Institute [63] and for *C. dubliniensis *[7] from GeneDB at the Wellcome Trust Sanger Institute [64]. The first assembly of the *C. parapsilosis *genome was downloaded from the Sanger Institute [65] and in-house gene annotations were called (as described in Fitzpatrick et al [8]). The resultant gene set contains 5,809 protein-coding genes. The *C. parapsilosis *genome was also automatically annotated by the Broad Institute [5], and we use these gene names where possible.

### Phylogenetic relationships

Phylogenetic relationships were determined using a supertree approach. All ten *Candida *genomes as well as two outgroups (*Saccharomyces cerevisiae *and *Candida glabrata*) were merged into a local Blast database. For a full descriptions of the methodology used please refer to Fitzpatrick et al [8].

### Homology pillars and genome editing

Sets of homologous genes are stored in CGOB's pillars (Figure 2). Pillars are the core data structures used to store homology assignments across all species [20]. All genes were integrated into homology pillars by performing an automated bi-directional best BLASTP hit (E- value cut-off of 10^-5^) strategy against *C. albicans *SC5314. A second round of automated searching merged singleton pillars using a BLASTP hit (E- value cut-off of 10^-5^) and synteny with at least one gene in an adjacent pillar. We then systematically manually edited CGOB by browsing along each *Candida *chromosome validating and refining homology pillars.

Several potential genes in the automatically called open reading frames sets are incomplete or "partial". We merged partial ORFs where possible, by aligning them against their complete orthologs from the other *Candida *genomes using Muscle [66]. The resultant alignments were manually checked and where appropriate, partial ORFs were merged and the resulting gene models were renamed, and added to CGOB's pillars. For completeness both the merged genes and the original partial ORFs have been retained in the CGOB Blast sequence database.

### Duplications

Genes that have arisen through tandem duplication were located using bl2seq from the NCBI suite of Blast executables. A tandem repeat was defined as adjacent genes with an E- value cut-off of 10^-10 ^with a highest scoring sequence pair (HSP) more than half the length of the shortest sequence. This approach filters out genes with similarity over short regions. Tandem genes that are evolving rapidly or have low sequence complexity may not be located using sequence similarity. We therefore programmed CGOB to compare tandem duplicates in all genomes, and used synteny to locate fast evolving tandems (or tandems with low complexity) in another genome.

Synonymous (d_S_) and nonsynonymous (d_N_) substitution rates for genes located in tandem clusters were estimated using the methods of Yang and Nielsen [67] as implemented in yn00 in the PAML suite [68].

To identify multigene families, every gene in a particular *Candida *proteome was searched against every other gene in its cognate genome. Genes with a BLASTP E- value less than 10^-30 ^and a HSP more than 60% the length of the shortest sequence were considered to be members of the same family, this is the same strategy used by Braun *et al *[31].

### Locating clusters of adjacent genes in metabolic pathways

Metabolic pathways for *C. albicans *SC5314 were downloaded from the *Candida *Genome Database [62]. The gene identifiers for each enzymatic step were mapped on CGOB. Clusters were defined as identifiers belonging to a particular metabolic pathway that lie within a contiguous window of 10 genes. The presence or absences of *C. albicans *SC5314 pathway homologs were then scored in the remaining nine *Candida *genomes.

For completeness we automatically inferred individual metabolic pathways for all *Candida *species using the KEGG automatic annotation server (KAAS) [69]. KAAS is based on reciprocally best BLAST similarity hits against all KEGG orthology (KO) groups of functionally related genes assigned in the KEGG GENES database. KAAS assigned each *Candida *gene a KO number and these were subsequently mapped to one of KEGG's reference metabolic pathways. All *Candida *KO identifiers were mapped onto CGOB and we searched for metabolic clusters as described above.

The significance of metabolic clusters was tested using simulations where gene order was randomized to give pseudogenomes. Both CGD and KEGG pathway components were mapped onto randomized genome data and scored as described above. This process was repeated 10000 times for each pathway in each *Candida *genome. Clusters are considered significant if the number of linked genes in the pseudogenome is less than that observed in the real genome 95% of the time.

## Abbreviations

**CGD**: *Candida *genome database; **CGOB**: Candida Gene Order Browser; **YGOB**: Yeast Gene Order Browser; **HSP: **highest scoring sequence pair; **KEGG**: Kyoto Encyclopedia of Genes and Genomes; **KCs**: KEGG clusters; **KAAS**: KEGG automatic annotation server; **KO**: KEGG orthology; **ORF: **open reading frame; **PIPKc**: Phosphatidylinositol Phosphate Kinase; **Nag**: *N*-acetylglucosamine; **HXK1**: hexokinase kinase; **DAC1***: *Nag-6-phosphate deacetylase; **bp**: basepairs; **d_S_**: synonymous substitution; **d_N_**: nonsynonymous substitution.

## Authors' contributions

DAF and GB were involved in the design phase. KPB developed and installed software. POG installed software. POG and DAF sourced homologs. DAF merged partial genes and manually curated homology columns. DAF and GB examined synteny, duplication and cluster data. DAF and GB drafted the manuscript. All authors read and approved the final manuscript.

## Supplementary Material

Additional file 1 **Merging partial open reading frames**. Section of alignment illustrating that the original automatically called gene sets contained partial open reading frames. In this example *LELG_01496 *and *LELG_01495 *from *L. elongisporus *are merged to give a new single gene (LELG_01496*). Click here for file

Additional file 2 **List of partial ORFs in datasets obtained from sequencing centers**. Merged genes all have a * suffix and are present in CGOB. Partial ORFs have been removed from CGOB pillars but are present in the CGOB Blast database. Click here for file

Additional file 3 **List of all tandem duplicates located by CGOB**. **a) **Clusters are labeled 1-502. Those with a d_N_/d_S _value > 1 are highlighted in red. Clusters displaying a ^ indicate that the initial BLAST search strategy failed to infer homology. Clusters displaying * indicate that there are intervening genes but they may be spurious gene models. Clusters displaying (INS) indicate that there is one intervening gene. **b) **List of tandem clusters with a d_N_/d_S _> 1. Click here for file

Additional file 4 **List of CGD pathways and the corresponding genes that display evidence of clustering in each *Candida *species**. Numbers in parenthesis refer to cluster numbers and are retained across species. Clusters with "sig" in parenthesis infer that the cluster is significantly better than randomized data. Click here for file

Additional file 5 **List of KEGG pathways and the corresponding genes that display evidence of clustering in each *Candida *species**. Clusters have been assigned numbers (KC) so it is possible to locate a cluster present in one species that is absent in another. Clusters denoted with a TD infer that the cluster has arisen through tandem duplication. Clusters that are significantly better than randomized data are highlighted with purple shading. Click here for file
