# Supplementary material for: Analysis of gene evolution and metabolic pathways using the Candida Gene Order Browser
Source: BMC Genomics. 2010 May 10;11:290. doi: 10.1186/1471-2164-11-290 (PMC2880306; doi:10.1186/1471-2164-11-290)
Supplement: Additional file 1 — Merging partial open reading frames. Section of alignment illustrating that the original automatically called gene sets contained partial open reading frames. In this example LELG_01496 and LELG_01495 from L. elongisporus are merged to give a new single gene (LELG_01496*). [file 1471-2164-11-290-S1.PDF]

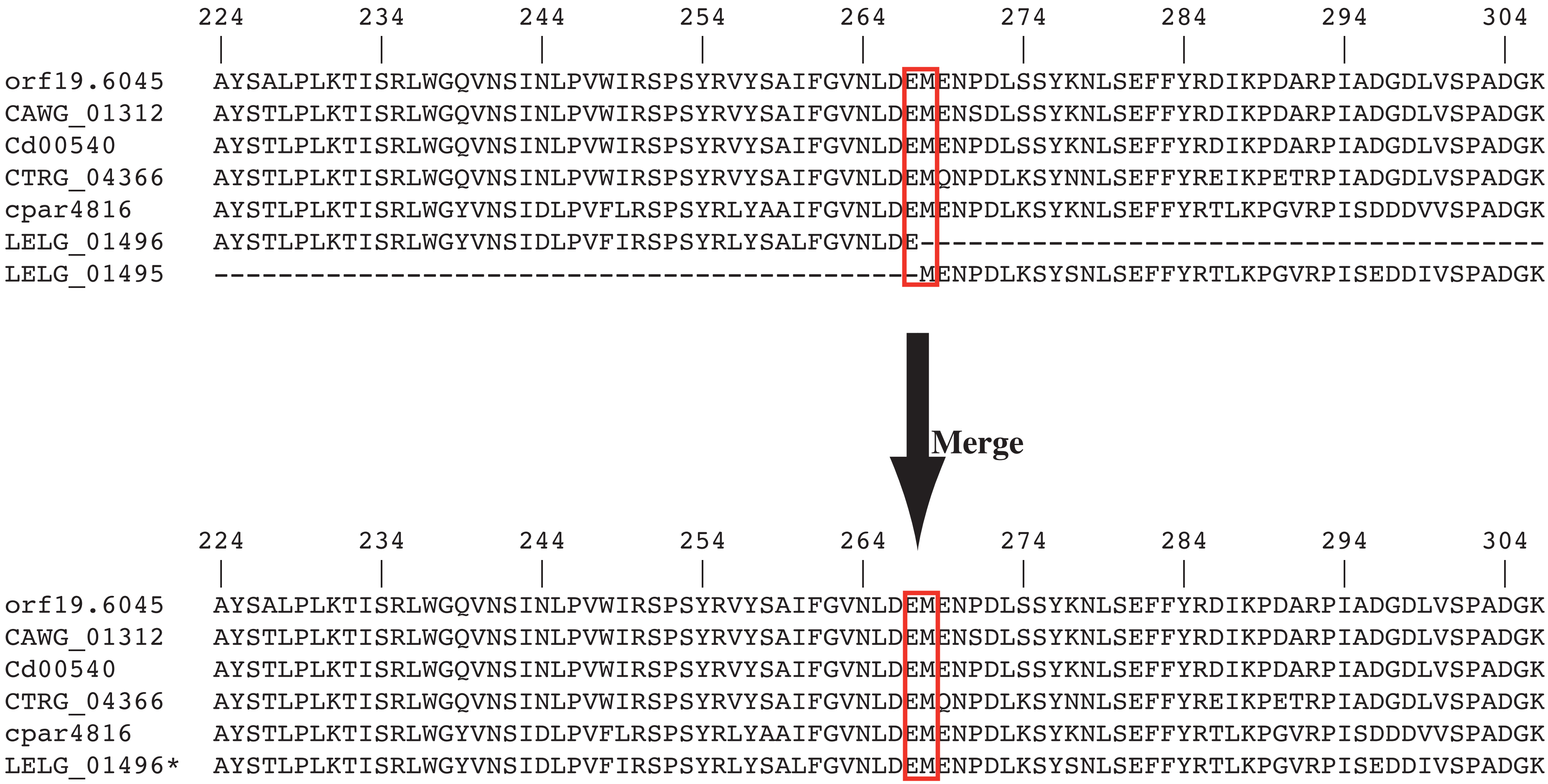
224 234 244 254 264 274 284 294 304
orf19.6045 AYSALPLKTISRLWGQVNSINLPVWIRSPSYRVYSAIFGVNLDEMENPDLSSYKNLSEFFYRDIKPDARPIADGDLVSPADGK
CAWG_01312 AYSTLPLKTISRLWGQVNSINLPVWIRSPSYRVYSAIFGVNLDEMENSDLSSYKNLSEFFYRDIKPDARPIADGDLVSPADGK
Cd00540 AYSTLPLKTISRLWGQVNSINLPVWIRSPSYRVYSAIFGVNLDEMENPDLSSYKNLSEFFYRDIKPDARPIADGDLVSPADGK
CTRG_04366 AYSTLPLKTISRLWGQVNSINLPVWIRSPSYRVYSAIFGVNLDEMQNPDLKSYNNLSEFFYREIKPETRPIADGDLVSPADGK
cpar4816 AYSTLPLKTISRLWGYVNSIDLPVFLRSPSYRLYAAIFGVNLDEMENPDLKSYKNLSEFFYRTLKPGVRPISDDDVVSPADGK
LELG_01496 AYSTLPLKTISRLWGYVNSIDLPVFIRSPSYRLYSALFGVNLDE---------------------------------------
LELG_01495 ---MENPDLKSYSNLSEFFYRTLKPGVRPISEDDIVSPADGK
Merge
224 234 244 254 264 274 284 294 304
orf19.6045 AYSALPLKTISRLWGQVNSINLPVWIRSPSYRVYSAIFGVNLDEMENPDLSSYKNLSEFFYRDIKPDARPIADGDLVSPADGK
CAWG_01312 AYSTLPLKTISRLWGQVNSINLPVWIRSPSYRVYSAIFGVNLDEMENSDLSSYKNLSEFFYRDIKPDARPIADGDLVSPADGK
Cd00540 AYSTLPLKTISRLWGQVNSINLPVWIRSPSYRVYSAIFGVNLDEMENPDLSSYKNLSEFFYRDIKPDARPIADGDLVSPADGK
CTRG_04366 AYSTLPLKTISRLWGQVNSINLPVWIRSPSYRVYSAIFGVNLDEMQNPDLKSYNNLSEFFYREIKPETRPIADGDLVSPADGK
cpar4816 AYSTLPLKTISRLWGYVNSIDLPVFLRSPSYRLYAAIFGVNLDEMENPDLKSYKNLSEFFYRTLKPGVRPISDDDVVSPADGK
LELG_01496* AYSTLPLKTISRLWGYVNSIDLPVFIRSPSYRLYSALFGVNLDEMENPDLKSYSNLSEFFYRTLKPGVRPISEDDIVSPADGK
