# Supplementary material for: Analysis of gene evolution and metabolic pathways using the Candida Gene Order Browser
Source: BMC Genomics. 2010 May 10;11:290. doi: 10.1186/1471-2164-11-290 (PMC2880306; doi:10.1186/1471-2164-11-290)
Supplement: Additional file 2 — List of partial ORFs in datasets obtained from sequencing centers. Merged genes all have a * suffix and are present in CGOB. Partial ORFs have been removed from CGOB pillars but are present in the CGOB Blast database. [file 1471-2164-11-290-S2.DOC]

**Additional File 2: List of partial proteins that have been merged**

| **Merged Protein** | **Partial 1** | **Partial 2** | **Partial 3** | **Partial 4** |
| --- | --- | --- | --- | --- |
| ***C. albicans WO-1*** |  |  |  |  |
| CAWG_00215* | CAWG_00215 | CAWG_00214 |  |  |
| CAWG_00362* | CAWG_00362 | CAWG_00361 |  |  |
| CAWG_00366* | CAWG_00366 | CAWG_00365 |  |  |
| CAWG_00643* | CAWG_00643 | CAWG_00642 |  |  |
| CAWG_00652* | CAWG_00652 | CAWG_00651 |  |  |
| CAWG_00666* | CAWG_00666 | CAWG_00665 |  |  |
| CAWG_00705* | CAWG_00705 | CAWG_00704 |  |  |
| CAWG_00778* | CAWG_00778 | CAWG_00777 |  |  |
| CAWG_00932* | CAWG_00932 | CAWG_00931 |  |  |
| CAWG_01142* | CAWG_01142 | CAWG_01141 | CAWG_01140 | CAWG_01139 |
| CAWG_01275* | CAWG_01275 | CAWG_01274 |  |  |
| CAWG_01398* | CAWG_01398 | CAWG_01397 |  |  |
| CAWG_01514* | CAWG_01514 | CAWG_01513 |  |  |
| CAWG_01528* | CAWG_01528 | CAWG_01527 |  |  |
| CAWG_01821* | CAWG_01821 | CAWG_01820 |  |  |
| CAWG_02005* | CAWG_02005 | CAWG_02004 |  |  |
| CAWG_02026* | CAWG_02026 | CAWG_02025 |  |  |
| CAWG_02153* | CAWG_02153 | CAWG_02152 | CAWG_02151 |  |
| CAWG_02485* | CAWG_02485 | CAWG_02484 |  |  |
| CAWG_02502* | CAWG_02502 | CAWG_02503 |  |  |
| CAWG_02722* | CAWG_02722 | CAWG_02721 | CAWG_02720 |  |
| CAWG_02983* | CAWG_02983 | CAWG_02982 |  |  |
| CAWG_03234* | CAWG_03234 | CAWG_03235 |  |  |
| CAWG_03289* | CAWG_03289 | CAWG_03288 |  |  |
| CAWG_03313* | CAWG_03313 | CAWG_03312 |  |  |
| CAWG_03432* | CAWG_03432 | CAWG_03431 |  |  |
| CAWG_03517* | CAWG_03517 | CAWG_03516 |  |  |
| CAWG_03685* | CAWG_03685 | CAWG_03684 | CAWG_03683 |  |
| CAWG_03770* | CAWG_03770 | CAWG_03769 |  |  |
| CAWG_03987* | CAWG_03987 | CAWG_03988 |  |  |
| CAWG_04507* | CAWG_04507 | CAWG_04506 |  |  |
| CAWG_04617* | CAWG_04617 | CAWG_04616 |  |  |
| CAWG_04779* | CAWG_04779 | CAWG_04778 |  |  |
| CAWG_04962* | CAWG_04962 | CAWG_04961 |  |  |
| CAWG_04967* | CAWG_04967 | CAWG_04966 |  |  |
| CAWG_05440* | CAWG_05440 | CAWG_05439 |  |  |
| CAWG_05619* | CAWG_05619 | CAWG_05618 |  |  |
| CAWG_05785* | CAWG_05785 | CAWG_05784 |  |  |
| CAWG_05935* | CAWG_05935 | CAWG_05934 |  |  |
| CAWG_02835* | CAWG_02835 | CAWG_02834 | CAWG_02833 |  |
| CAWG_04087* | CAWG_04087 | CAWG_04086 | CAWG_04085 |  |
| CAWG_05446* | CAWG_05446 | CAWG_05445 |  |  |
| ***C. tropicalis*** |  |  |  |  |
| CTRG_00055* | CTRG_00055 | CTRG_00054 | CTRG_00053 |  |
| CTRG_00189* | CTRG_00189 | CTRG_00188 |  |  |
| CTRG_00217* | CTRG_00217 | CTRG_00216 |  |  |
| CTRG_00237* | CTRG_00237 | CTRG_00236 |  |  |
| CTRG_00464* | CTRG_00464 | CTRG_00461 |  |  |
| CTRG_00473* | CTRG_00473 | CTRG_00472 |  |  |
| CTRG_00840* | CTRG_00840 | CTRG_00839 |  |  |
| CTRG_01059* | CTRG_01059 | CTRG_01058 | CTRG_01057 |  |
| CTRG_01471* | CTRG_01471 | CTRG_01470 |  |  |
| CTRG_01477* | CTRG_01477 | CTRG_01476 |  |  |
| CTRG_01479* | CTRG_01479 | CTRG_01478 |  |  |
| CTRG_01493* | CTRG_01493 | CTRG_01492 |  |  |
| CTRG_01516* | CTRG_01516 | CTRG_01515 |  |  |
| CTRG_01598* | CTRG_01598 | CTRG_01597 |  |  |
| CTRG_01693* | CTRG_01693 | CTRG_01692 |  |  |
| CTRG_01703* | CTRG_01703 | CTRG_01702 |  |  |
| CTRG_01851* | CTRG_01851 | CTRG_01850 |  |  |
| CTRG_01911* | CTRG_01911 | CTRG_01910 |  |  |
| CTRG_02088* | CTRG_02088 | CTRG_02087 |  |  |
| CTRG_02145* | CTRG_02145 | CTRG_02144 |  |  |
| CTRG_02186* | CTRG_02186 | CTRG_02185 |  |  |
| CTRG_02194* | CTRG_02194 | CTRG_02193 |  |  |
| CTRG_02197* | CTRG_02197 | CTRG_02196 |  |  |
| CTRG_02491* | CTRG_02491 | CTRG_02490 |  |  |
| CTRG_02792* | CTRG_02793 | CTRG_02792 | CTRG_02791 | CTRG_02790 |
| CTRG_02858* | CTRG_02858 | CTRG_02857 |  |  |
| CTRG_03063* | CTRG_03063 | CTRG_03062 |  |  |
| CTRG_03211* | CTRG_03211 | CTRG_03210 |  |  |
| CTRG_03213* | CTRG_03213 | CTRG_03212 |  |  |
| CTRG_03283* | CTRG_03283 | CTRG_03281 |  |  |
| CTRG_03325* | CTRG_03325 | CTRG_03324 |  |  |
| CTRG_03366* | CTRG_03366 | CTRG_03365 |  |  |
| CTRG_03511* | CTRG_03511 | CTRG_03510 |  |  |
| CTRG_03738* | CTRG_03738 | CTRG_03737 |  |  |
| CTRG_03777* | CTRG_03777 | CTRG_03776 |  |  |
| CTRG_03858* | CTRG_03858 | CTRG_03857 |  |  |
| CTRG_04181* | CTRG_04181 | CTRG_04180 |  |  |
| CTRG_04218* | CTRG_04218 | CTRG_04217 |  |  |
| CTRG_04239* | CTRG_04239 | CTRG_04238 |  |  |
| CTRG_04369* | CTRG_04369 | CTRG_04368 |  |  |
| CTRG_04450* | CTRG_04450 | CTRG_04449 |  |  |
| CTRG_04522* | CTRG_04522 | CTRG_04521 |  |  |
| CTRG_04539* | CTRG_04539 | CTRG_04538 |  |  |
| CTRG_04584* | CTRG_04584 | CTRG_04583 |  |  |
| CTRG_04675* | CTRG_04675 | CTRG_04674 |  |  |
| CTRG_05021* | CTRG_05021 | CTRG_05020 |  |  |
| CTRG_05023* | CTRG_05023 | CTRG_05022 |  |  |
| CTRG_05025* | CTRG_05025 | CTRG_05024 |  |  |
| CTRG_05082* | CTRG_05082 | CTRG_05081 |  |  |
| CTRG_05217* | CTRG_05217 | CTRG_05216 |  |  |
| CTRG_05371* | CTRG_05371 | CTRG_05370 |  |  |
| CTRG_05552* | CTRG_05552 | CTRG_05551 |  |  |
| CTRG_05638* | CTRG_05638 | CTRG_05637 |  |  |
| CTRG_05640* | CTRG_05640 | CTRG_05639 |  |  |
| CTRG_05684* | CTRG_05684 | CTRG_05683 |  |  |
| CTRG_05946* | CTRG_05946 | CTRG_05945 |  |  |
| CTRG_06155* | CTRG_06155 | CTRG_06154 |  |  |
| ***C. parapsilosis*** |  |  |  |  |
| cpar1145* / CPAG_00165* | cpar1145/CPAG_00165 | cpar1966/CPAG_00086 |  |  |
| cpar2838* /CPAG_00667* | cpar2838/CPAG_00667 | cpar1170 |  |  |
| cpar1300* /CPAG_02361* | cpar1300/CPAG_02361 | cpar3359/CPAG_00862 |  |  |
| cpar1568* /CPAG_03061* | cpar1568/CPAG_03061 | cpar4269 |  |  |
| cpar1569* /CPAG_01801 | cpar1569/CPAG_01801 | cpar4740/CPAG_02629 |  |  |
| cpar4089* /CPAG_05554 | cpar4089/CPAG_05554 | cpar4088/CPAG_05553 |  |  |
| cpar4566* | cpar4566 | cpar4805 |  |  |
| cpar4632* /CPAG_03258* | cpar4632/CPAG_03258 | cpar4631/CPAG_03257 |  |  |
| cpar4926* /CPAG_04948* | cpar4926/CPAG_04948 | cpar4925/CPAG_04947 |  |  |
| cpar5089* /CPAG_02908* | cpar5089/CPAG_02908 | cpar5088 |  |  |
| cpar5512* /CPAG_00677* | cpar5512/CPAG_00677 | cpar5511 |  |  |
| cpar4001* /CPAG_04486* | cpar4001 | cpar5165 |  |  |
| cpar5166* /CPAG_02753* | cpar5166 | cpar2335 |  |  |
| cpar488* /CPAG_00219* | cpar488 | cpar4302 |  |  |
| ***L. elongisporus*** |  |  |  |  |
| LELG_00009* | LELG_00009 | LELG_00008 |  |  |
| LELG_00023* | LELG_00023 | LELG_00022 |  |  |
| LELG_00026* | LELG_00026 | LELG_00025 |  |  |
| LELG_00040* | LELG_00040 | LELG_00038 |  |  |
| LELG_00052* | LELG_00052 | LELG_00051 |  |  |
| LELG_00288* | LELG_00288 | LELG_00287 |  |  |
| LELG_00300* | LELG_00300 | LELG_00298 |  |  |
| LELG_00324* | LELG_00324 | LELG_00323 |  |  |
| LELG_00612* | LELG_00612 | LELG_00610 |  |  |
| LELG_00619* | LELG_00619 | LELG_00618 |  |  |
| LELG_00665* | LELG_00665 | LELG_00664 | LELG_00663 |  |
| LELG_00704* | LELG_00704 | LELG_00703 |  |  |
| LELG_00950 | LELG_00950 | LELG_00949 |  |  |
| LELG_01108* | LELG_01108 | LELG_01109 |  |  |
| LELG_01385* | LELG_01385 | LELG_01384 |  |  |
| LELG_01387* | LELG_01387 | LELG_01388 |  |  |
| LELG_01425* | LELG_01425 | LELG_01424 |  |  |
| LELG_01440* | LELG_01440 | LELG_01439 |  |  |
| LELG_01496* | LELG_01496 | LELG_01495 |  |  |
| LELG_01591* | LELG_01591 | LELG_01590 |  |  |
| LELG_01741* | LELG_01741 | LELG_01740 |  |  |
| LELG_01841* | LELG_01841 | LELG_01840 |  |  |
| LELG_01851* | LELG_01851 | LELG_01850 |  |  |
| LELG_01888* | LELG_01888 | LELG_01887 | LELG_01886 |  |
| LELG_01943* | LELG_01943 | LELG_01941 |  |  |
| LELG_01969* | LELG_01969 | LELG_01968 | LELG_01967 | LELG_01966 |
| LELG_02348* | LELG_02348 | LELG_02347 |  |  |
| LELG_02487* | LELG_02487 | LELG_02486 |  |  |
| LELG_02653* | LELG_02653 | LELG_02652 |  |  |
| LELG_02663* | LELG_02663 | LELG_02662 |  |  |
| LELG_02665* | LELG_02665 | LELG_02664 |  |  |
| LELG_02817* | LELG_02817 | LELG_02816 |  |  |
| LELG_02845* | LELG_02845 | LELG_02844 |  |  |
| LELG_02856* | LELG_02856 | LELG_02855 |  |  |
| LELG_02892* | LELG_02892 | LELG_02891 |  |  |
| LELG_03026* | LELG_03026 | LELG_03025 |  |  |
| LELG_03046* | LELG_03046 | LELG_03044 |  |  |
| LELG_03087* | LELG_03087 | LELG_03086 |  |  |
| LELG_03099* | LELG_03099 | LELG_03098 |  |  |
| LELG_03175* | LELG_03175 | LELG_03174 |  |  |
| LELG_03372* | LELG_03372 | LELG_03371 |  |  |
| LELG_03456* | LELG_03456 | LELG_03455 |  |  |
| LELG_03508* | LELG_03508 | LELG_03507 |  |  |
| LELG_03757* | LELG_03757 | LELG_03756 |  |  |
| LELG_03856* | LELG_03856 | LELG_03855 |  |  |
| LELG_03898* | LELG_03898 | LELG_03897 |  |  |
| LELG_04062* | LELG_04062 | LELG_04061 |  |  |
| LELG_04143* | LELG_04143 | LELG_04142 |  |  |
| LELG_04160* | LELG_04160 | LELG_04159 | LELG_04158 | LELG_04157 |
| LELG_04177* | LELG_04177 | LELG_04176 |  |  |
| LELG_04189* | LELG_04189 | LELG_04188 |  |  |
| LELG_04323* | LELG_04323 | LELG_04322 |  |  |
| LELG_04326* | LELG_04326 | LELG_04324 |  |  |
| LELG_04351* | LELG_04351 | LELG_04350 |  |  |
| LELG_04373* | LELG_04373 | LELG_04372 | LELG_04371 | LELG_04370 |
| LELG_04517* | LELG_04517 | LELG_04516 |  |  |
| LELG_04542* | LELG_04542 | LELG_04541 |  |  |
| LELG_04614* | LELG_04614 | LELG_04613 |  |  |
| LELG_04779* | LELG_04779 | LELG_04778 |  |  |
| LELG_04832* | LELG_04832 | LELG_04831 |  |  |
| LELG_04835* | LELG_04835 | LELG_04834 |  |  |
| LELG_04905* | LELG_04905 | LELG_04904 |  |  |
| LELG_04916* | LELG_04916 | LELG_04915 |  |  |
| LELG_04918* | LELG_04918 | LELG_04917 |  |  |
| LELG_04929* | LELG_04929 | LELG_04928 |  |  |
| LELG_04997* | LELG_04997 | LELG_04996 |  |  |
| LELG_05022* | LELG_05022 | LELG_05021 |  |  |
| LELG_05078* | LELG_05078 | LELG_05077 |  |  |
| LELG_05095* | LELG_05095 | LELG_05094 |  |  |
| LELG_05150* | LELG_05150 | LELG_05149 |  |  |
| LELG_05412* | LELG_05412 | LELG_05411 |  |  |
| LELG_05491* | LELG_05491 | LELG_05490 |  |  |
| LELG_05578* | LELG_05578 | LELG_05577 |  |  |
| LELG_02183* | LELG_02184 | LELG_02183 | LELG_02182 | LELG_02181 |
| LELG_03650* | LELG_03650 | LELG_03649 | LELG_03648 |  |
| LELG_03454* | LELG_03454 | LELG_03453 |  |  |
| LELG_04771* | LELG_04771 | LELG_04770 |  |  |
| LELG_05584* | LELG_05584 | LELG_05583 |  |  |
| LELG_00404* | LELG_00404 | LELG_00403 |  |  |
| LELG_04803* | LELG_04803 | LELG_04802 |  |  |
| LELG_00740* | LELG_00740 | LELG_00739 |  |  |
| ***D. hansenii*** |  |  |  |  |
| DEHA0C18546g* | DEHA0C18546g | DEHA0C18535g |  |  |
| DEHA0E02046g* | DEHA0E02046g | DEHA0E02024g |  |  |
| DEHA0F09361g* | DEHA0F09361g | DEHA0F09350g |  |  |
| DEHA0G16390g* | DEHA0G16390g | DEHA0G16368g |  |  |
| DEHA0D18854g* | DEHA0D18854g | DEHA0D18876g |  |  |
| DEHA0C18579g* | DEHA0C18568g | DEHA0C18579g |  |  |
| ***P. stipitis*** |  |  |  |  |
| PICST_19389* | PICST_19389 | PICST_60166 |  |  |
| PICST_39246* | PICST_39246 | PICST_28236 |  |  |
| PICST_48188* | PICST_48188 | PICST_36661 |  |  |
| PICST_49751* | PICST_49751 | PICST_36940 |  |  |
| PICST_67602* | PICST_67602 | PICST_17868 |  |  |
| PICST_67905* | PICST_67905 | PICST_67906 |  |  |
| ***C. guillermondii*** |  |  |  |  |
| PGUG_00096* | PGUG_00096 | PGUG_00095 |  |  |
| PGUG_00276* | PGUG_00276 | PGUG_00275 |  |  |
| PGUG_00422* | PGUG_00422 | PGUG_00421 |  |  |
| PGUG_00451* | PGUG_00451 | PGUG_00450 |  |  |
| PGUG_00510* | PGUG_00510 | PGUG_00509 |  |  |
| PGUG_00586* | PGUG_00586 | PGUG_00585 |  |  |
| PGUG_00633* | PGUG_00633 | PGUG_00632 | PGUG_00631 |  |
| PGUG_00684* | PGUG_00684 | PGUG_00683 |  |  |
| PGUG_00786* | PGUG_00786 | PGUG_00785 |  |  |
| PGUG_00846* | PGUG_00846 | PGUG_00845 |  |  |
| PGUG_00886* | PGUG_00886 | PGUG_00885 |  |  |
| PGUG_00963* | PGUG_00963 | PGUG_00962 |  |  |
| PGUG_01004* | PGUG_01004 | PGUG_01003 |  |  |
| PGUG_01107* | PGUG_01107 | PGUG_01105 |  |  |
| PGUG_01131* | PGUG_01131 | PGUG_01130 | PGUG_01129 |  |
| PGUG_01236* | PGUG_01236 | PGUG_01235 |  |  |
| PGUG_01246* | PGUG_01246 | PGUG_01245 |  |  |
| PGUG_01251* | PGUG_01251 | PGUG_01250 |  |  |
| PGUG_01461* | PGUG_01461 | PGUG_01462 |  |  |
| PGUG_01472* | PGUG_01472 | PGUG_01470 |  |  |
| PGUG_01583* | PGUG_01583 | PGUG_01582 | PGUG_01580 |  |
| PGUG_01661* | PGUG_01661 | PGUG_01660 |  |  |
| PGUG_01718* | PGUG_01718 | PGUG_01717 |  |  |
| PGUG_01768* | PGUG_01768 | PGUG_01767 |  |  |
| PGUG_01770* | PGUG_01770 | PGUG_01769 |  |  |
| PGUG_01893* | PGUG_01893 | PGUG_01892 |  |  |
| PGUG_02048* | PGUG_02048 | PGUG_02047 |  |  |
| PGUG_02073* | PGUG_02073 | PGUG_02072 |  |  |
| PGUG_02184* | PGUG_02184 | PGUG_02183 |  |  |
| PGUG_02246* | PGUG_02246 | PGUG_02245 |  |  |
| PGUG_02256* | PGUG_02256 | PGUG_02255 |  |  |
| PGUG_02394* | PGUG_02394 | PGUG_02393 |  |  |
| PGUG_02453* | PGUG_02453 | PGUG_02452 | PGUG_02451 |  |
| PGUG_02455* | PGUG_02455 | PGUG_02454 |  |  |
| PGUG_02637* | PGUG_02637 | PGUG_02636 |  |  |
| PGUG_02840* | PGUG_02840 | PGUG_02839 |  |  |
| PGUG_02881* | PGUG_02881 | PGUG_02880 |  |  |
| PGUG_03000* | PGUG_03000 | PGUG_02999 | PGUG_02998 |  |
| PGUG_03037* | PGUG_03037 | PGUG_03036 | PGUG_03035 |  |
| PGUG_03104* | PGUG_03104 | PGUG_03103 | PGUG_03102 |  |
| PGUG_03252* | PGUG_03252 | PGUG_03251 |  |  |
| PGUG_03671* | PGUG_03671 | PGUG_03670 |  |  |
| PGUG_03682* | PGUG_03682 | PGUG_03681 |  |  |
| PGUG_03746* | PGUG_03746 | PGUG_03745 |  |  |
| PGUG_03865* | PGUG_03865 | PGUG_03863 |  |  |
| PGUG_03876* | PGUG_03876 | PGUG_03875 |  |  |
| PGUG_03912* | PGUG_03912 | PGUG_03911 |  |  |
| PGUG_04255* | PGUG_04255 | PGUG_04254 |  |  |
| PGUG_04337* | PGUG_04337 | PGUG_04336 |  |  |
| PGUG_04416* | PGUG_04416 | PGUG_04415 |  |  |
| PGUG_04450* | PGUG_04450 | PGUG_04449 |  |  |
| PGUG_04935* | PGUG_04935 | PGUG_04934 |  |  |
| PGUG_04962* | PGUG_04962 | PGUG_04961 |  |  |
| PGUG_05070* | PGUG_05070 | PGUG_05069 |  |  |
| PGUG_05268* | PGUG_05268 | PGUG_05269 |  |  |
| PGUG_05553* | PGUG_05553 | PGUG_05552 |  |  |
| PGUG_05588* | PGUG_05588 | PGUG_05587 | PGUG_05586 |  |
| PGUG_05605* | PGUG_05605 | PGUG_05604 |  |  |
| PGUG_05739* | PGUG_05739 | PGUG_05738 |  |  |
| PGUG_04486* | PGUG_04486 | PGUG_04485 |  |  |
| PGUG_04489* | PGUG_04489 | PGUG_04488 |  |  |
| PGUG_00751* | PGUG_00751 | PGUG_00750 |  |  |
| PGUG_01233* | PGUG_01233 | PGUG_01232 |  |  |
| PGUG_01766* | PGUG_01766 | PGUG_01765 |  |  |
| PGUG_03674* | PGUG_03674 | PGUG_03673 |  |  |
| PGUG_00709* | PGUG_00710 | PGUG_00709 | PGUG_00708 | PGUG_00707 |
| ***C. lusitaniae*** |  |  |  |  |
| CLUG_00197* | CLUG_00197 | CLUG_00196 |  |  |
| CLUG_00290* | CLUG_00290 | CLUG_00289 |  |  |
| CLUG_00309* | CLUG_00309 | CLUG_00308 |  |  |
| CLUG_00399* | CLUG_00399 | CLUG_00398 |  |  |
| CLUG_00662* | CLUG_00662 | CLUG_00661 |  |  |
| CLUG_00665* | CLUG_00665 | CLUG_00664 |  |  |
| CLUG_00930* | CLUG_00930 | CLUG_00929 |  |  |
| CLUG_00978* | CLUG_00978 | CLUG_00977 |  |  |
| CLUG_00986* | CLUG_00986 | CLUG_00985 |  |  |
| CLUG_01027* | CLUG_01027 | CLUG_01026 |  |  |
| CLUG_01127* | CLUG_01127 | CLUG_01126 |  |  |
| CLUG_01158* | CLUG_01158 | CLUG_01157 |  |  |
| CLUG_01188* | CLUG_01188 | CLUG_01187 |  |  |
| CLUG_01224* | CLUG_01224 | CLUG_01223 |  |  |
| CLUG_01295* | CLUG_01295 | CLUG_01294 |  |  |
| CLUG_01222* | CLUG_01222 | CLUG_01221 |  |  |
| CLUG_01429* | CLUG_01429 | CLUG_01428 |  |  |
| CLUG_01470* | CLUG_01470 | CLUG_01468 |  |  |
| CLUG_01499* | CLUG_01499 | CLUG_01498 |  |  |
| CLUG_01587* | CLUG_01587 | CLUG_01586 |  |  |
| CLUG_01656* | CLUG_01656 | CLUG_01655 |  |  |
| CLUG_01718* | CLUG_01718 | CLUG_01717 |  |  |
| CLUG_01732* | CLUG_01732 | CLUG_01731 | CLUG_01730 |  |
| CLUG_02253* | CLUG_02253 | CLUG_02252 |  |  |
| CLUG_02467* | CLUG_02467 | CLUG_02466 |  |  |
| CLUG_02532* | CLUG_02532 | CLUG_02531 |  |  |
| CLUG_02557* | CLUG_02557 | CLUG_02555 |  |  |
| CLUG_02586* | CLUG_02586 | CLUG_02585 |  |  |
| CLUG_02795* | CLUG_02795 | CLUG_02793 | CLUG_02792 | CLUG_02791 |
| CLUG_02830* | CLUG_02830 | CLUG_02828 |  |  |
| CLUG_01336* | CLUG_01336 | CLUG_01335 |  |  |
| CLUG_03061* | CLUG_03061 | CLUG_03060 | CLUG_03059 |  |
| CLUG_03143* | CLUG_03143 | CLUG_03142 |  |  |
| CLUG_03182* | CLUG_03182 | CLUG_03181 |  |  |
| CLUG_03187* | CLUG_03187 | CLUG_03186 |  |  |
| CLUG_03406* | CLUG_03406 | CLUG_03410 |  |  |
| CLUG_03658 | CLUG_03658 | CLUG_03657 |  |  |
| CLUG_03808* | CLUG_03808 | CLUG_03807 |  |  |
| CLUG_03822* | CLUG_03822 | CLUG_03821 |  |  |
| CLUG_03866* | CLUG_03866 | CLUG_03865 |  |  |
| CLUG_03943* | CLUG_03943 | CLUG_03942 | CLUG_03941 |  |
| CLUG_03974* | CLUG_03974 | CLUG_03973 | CLUG_03972 |  |
| CLUG_04191* | CLUG_04191 | CLUG_04190 |  |  |
| CLUG_04526* | CLUG_04526 | CLUG_04525 |  |  |
| CLUG_04570* | CLUG_04570 | CLUG_04568 |  |  |
| CLUG_04578* | CLUG_04578 | CLUG_04575 |  |  |
| CLUG_04580* | CLUG_04580 | CLUG_04579 |  |  |
| CLUG_04584* | CLUG_04584 | CLUG_04583 |  |  |
| CLUG_04637* | CLUG_04637 | CLUG_04636 |  |  |
| CLUG_04702* | CLUG_04702 | CLUG_04701 |  |  |
| CLUG_04739* | CLUG_04739 | CLUG_04738 |  |  |
| CLUG_04779* | CLUG_04779 | CLUG_04780 |  |  |
| CLUG_04833* | CLUG_04833 | CLUG_04832 |  |  |
| CLUG_04970* | CLUG_04970 | CLUG_04969 |  |  |
| CLUG_05065* | CLUG_05065 | CLUG_05063 |  |  |
| CLUG_05076* | CLUG_05076 | CLUG_05075 |  |  |
| CLUG_05208* | CLUG_05207 | CLUG_05208 |  |  |
| CLUG_05645* | CLUG_05645 | CLUG_05644 |  |  |
| CLUG_05890* | CLUG_05890 | CLUG_05889 |  |  |
| CLUG_03630* | CLUG_03630 | CLUG_03629 |  |  |
| CLUG_01004* | CLUG_01004 | CLUG_01003 |  |  |
| CLUG_01025* | CLUG_01025 | CLUG_01024 | CLUG_01023 |  |
| CLUG_04611* | CLUG_04612 | CLUG_04611 | CLUG_04610 | CLUG_04609 |
