# Supplementary material for: Analysis of gene evolution and metabolic pathways using the Candida Gene Order Browser
Source: BMC Genomics. 2010 May 10;11:290. doi: 10.1186/1471-2164-11-290 (PMC2880306; doi:10.1186/1471-2164-11-290)
Supplement: Additional file 4 — List of CGD pathways and the corresponding genes that display evidence of clustering in each Candida species. Numbers in parenthesis refer to cluster numbers and are retained across species. Clusters with "sig" in parenthesis infer that the cluster is significantly better than randomized data. [file 1471-2164-11-290-S4.DOC]

**Additional file 4: CGD pathways and corresponding genes that display evidence of gene clustering.**

| **Pathway** | **Clustered Genes** |
| --- | --- |
| superpathway of histidine, purine and pyrimidine biosynthesis | orf19.4827 orf19.4836 ^(1)^ |
|  | orf19.5779 orf19.5789 orf19.5801 ^(2)^ |
|  | orf19.5505 orf19.6699 ^(3)^ |
| aerobic respiration (cyanide insensitive) | orf19.4773 orf19.4774 |
| aerobic respiration (cyanide sensitive) ^(sig)^ | orf19.1467 orf19.1471 orf19.1480 |
| 2-keto glutarate dehydrogenase complex ^(sig)^ | orf19.6126 orf19.6127 |
| N-acetylglucosamine degradation ^(sig)^ | orf19.2154 orf19.2156 orf19.2157 |
| methylglyoxal pathway | orf19.5805 orf19.5806 |
| biotin biosynthesis ^(sig)^ | orf19.2587 orf19.2590 orf19.2591 orf19.2593 |
| tetrapyrrole biosynthesis | orf19.1742 orf19.1744 |
| pantothenate and coenzyme A biosynthesis | orf19.4005 orf19.4006 |
| arginine degradation (arginase pathway) ^(sig)^ | orf19.5641 orf19.5650 |
| galactose degradation ^(sig)^ | orf19.3670 orf19.3672 orf19.3674 orf19.3675 |
| tRNA charging pathway ^(sig)^ | orf19.2382 orf19.2387 ^(1)^ |
|  | orf19.2407 orf19.2415 ^(2)^ |
|  | orf19.2560 orf19.2573 ^(3)^ |
|  | orf19.3950 orf19.3955 ^(4)^ |
|  | orf19.6698 orf19.6701 orf19.6702 ^(5)^ |
|  | orf19.7057 orf19.7064 ^(6)^ |
| fatty acid oxidation pathway | orf19.1652 orf19.1655 ^(1)^ |
|  | orf19.6443 orf19.6445 ^(2)^ |
| glutathione-glutaredoxin redox reactions ^(sig)^ | orf19.356 orf19.359 orf19.6947 ^(1)^ |
|  | orf19.85 orf19.86 orf19.87 ^(2)^ |
| Fermentation/isoleucine & phenylalanine degradation | orf19.4504 orf19.4505 |
| removal of superoxide radicals | orf19.2060 orf19.2062 |

| **Pathway** | **Clustered Genes** |
| --- | --- |
| superpathway of histidine, purine and pyrimidine biosynthesis | CAWG_00458 CAWG_00466 ^(1)^ |
|  | CAWG_04068 CAWG_04076 CAWG_04083 ^(2)^ |
|  | CAWG_05696 CAWG_05703 ^(3)^ |
| aerobic respiration (cyanide insensitive) | CAWG_00513 CAWG_00514 |
| aerobic respiration (cyanide sensitive) ^(sig)^ | CAWG_03931 CAWG_03934 CAWG_03941 |
| 2-keto glutarate dehydrogenase complex ^(sig)^ | CAWG_02038 CAWG_02039 |
| N-acetylglucosamine degradation ^(sig)^ | CAWG_04921 CAWG_04922 CAWG_04923 |
| methylglyoxal pathway | CAWG_04064 CAWG_04065 |
| biotin biosynthesis ^(sig)^ | CAWG_01544 CAWG_01545 CAWG_01546 CAWG_01547 |
| tetrapyrrole biosynthesis | CAWG_06105 CAWG_06107 |
| pantothenate and coenzyme A biosynthesis | CAWG_04879 CAWG_04880 |
| galactose degradation ^(sig)^ | CAWG_01165 CAWG_01166 CAWG_01168 CAWG_01170 |
| tRNA charging pathway ^(sig)^ | CAWG_01670 CAWG_01675 ^(1)^  CAWG_01647 CAWG_01653 ^(2)^  CAWG_01519 CAWG_01526 ^(3)^ |
|  | CAWG_04833 CAWG_04837 ^(4)^ |
|  | CAWG_05695 CAWG_05697 CAWG_05698 ^(5)^  CAWG_05411 CAWG_05418 ^(6)^ |
| fatty acid oxidation pathway | CAWG_02521 CAWG_02524 ^(1)^ |
|  | CAWG_02154 CAWG_02156 ^(2)^ |
| glutathione-glutaredoxin redox reactions ^(sig)^ | CAWG_02636 CAWG_02638 ^(1)^ |
|  | CAWG_05273 CAWG_05274 CAWG_05275* ^(2)^ |
| Fermentation/isoleucine & phenylalanine degradation | CAWG_04203 CAWG_04204 |
| removal of superoxide radicals | CAWG_03845 CAWG_03847 |

| **Pathway** | **Clustered Genes** |
| --- | --- |
| superpathway of histidine, purine and pyrimidine biosynthesis | Cd36_09080 Cd36_09160 |
|  | Cd36_17780 Cd36_17860 Cd36_17930 ^(2)^ |
|  | Cd36_73290 Cd36_73390 ^(3)^ |
| aerobic respiration (cyanide insensitive) | Cd36_08630 Cd36_08640 |
| aerobic respiration (cyanide sensitive) ^(sig)^ | Cd36_16440 Cd36_16480 Cd36_16550 |
| 2-keto glutarate dehydrogenase complex ^(sig)^ | Cd36_33030 Cd36_33050 |
| N-acetylglucosamine degradation ^(sig)^ | Cd36_65510 Cd36_65540 Cd36_65550 |
| methylglyoxal pathway | Cd36_17740 Cd36_17750 |
| tetrapyrrole biosynthesis | Cd36_24400 Cd36_24420 |
| pantothenate and coenzyme A biosynthesis | Cd36_54730 Cd36_54740 |
| arginine degradation ^(sig)^ | Cd36_40230 Cd36_40310 |
| galactose degradation ^(sig)^ | Cd36_01990 Cd36_02000 Cd36_02020 Cd36_02030 |
| tRNA charging pathway ^(sig)^ | Cd36_28500 Cd36_28550 ^(1)^  Cd36_28230 Cd36_28310 ^(2)^  Cd36_26670 Cd36_26780 ^(3)^ |
|  | Cd36_54270 Cd36_54320 ^(4)^ |
|  | Cd36_73270 Cd36_73280 Cd36_73300 ^(5)^  Cd36_70040 Cd36_70110 ^(6)^ |
| fatty acid oxidation pathway | Cd36_34260 Cd36_34280 ^(2)^ |
| glutathione-glutaredoxin redox reactions ^(sig)^ | Cd36_83590 Cd36_83610 Cd36_83710 ^(1)^ |
|  | Cd36_60850 Cd36_60860 Cd36_60870 ^(2)^ |
| Removal of superoxide radicals | Cd36_15610 Cd36_15620 |

| **Pathway** | **Clustered Genes** |
| --- | --- |
| superpathway of histidine, purine and pyrimidine biosynthesis | CTRG_03386 CTRG_03394 ^(1)^ |
|  | CTRG_01309 CTRG_01316 CTRG_01327 ^(2) *^ |
| aerobic respiration (cyanide sensitive) ^(sig)^ | CTRG_01250 CTRG_01253 CTRG_01263 |
| 2-keto glutarate dehydrogenase complex ^(sig)^ | CTRG_03071 CTRG_03072 |
| N-acetylglucosamine degradation ^(sig)^ | CTRG_03726 CTRG_03727 CTRG_03728 |
| methylglyoxal pathway | CTRG_01342 CTRG_01343 |
| biotin biosynthesis ^(sig)^ | CTRG_05852 CTRG_05853 CTRG_05854 |
| NAD salvage pathway | CTRG_05150 CTRG_05154 |
| tetrapyrrole biosynthesis | CTRG_02031 CTRG_02033 |
| pantothenate and coenzyme A biosynthesis | CTRG_06121 CTRG_06122 |
| lipid-linked oligosaccharide biosynthesis | CTRG_02748 CTRG_02752 * |
| lysine biosynthesis ^(sig)^ | CTRG_04682 CTRG_04686 |
| galactose degradation ^(sig)^ | CTRG_04617 CTRG_04618 CTRG_04620 CTRG_04621 |
| tRNA charging pathway ^(sig)^ | CTRG_00760 CTRG_00765 ^(1)^ |
|  | CTRG_00783 CTRG_00789 ^(2)^ |
|  | CTRG_00846 CTRG_00853 ^(3)^ |
|  | CTRG_06069 CTRG_06073 ^(4)^  CTRG_05158 CTRG_05159 ^(5)^ |
| fatty acid oxidation pathway | CTRG_02374 CTRG_02377 ^(1)^ |
|  | CTRG_03043 CTRG_03045 ^(2)^ |
| glutathione-glutaredoxin redox reactions ^(sig)^ | CTRG_02946 CTRG_02947 CTRG_02948 ^(2)^ |
| Acrylonitrile degradation | CTRG_04984 CTRG_04986 |

| **Pathway** | **Clustered Genes** |
| --- | --- |
| superpathway of histidine, purine and pyrimidine biosynthesis | cpar4744(CPAG_02633) cpar4752(CPAG_02641) ^(1)*^ |
|  | cpar2291(CPAG_00540) cpar2298(CPAG_00547) ^(2)^ |
| aerobic respiration (cyanide insensitive) | cpar93(CPAG_04301) cpar94(CPAG_04302) |
| aerobic respiration (cyanide sensitive) | cpar2383(CPAG_01949) cpar2386(CPAG_01952) |
| N-acetylglucosamine degradation ^(sig)^ | cpar10(CPAG_04221) cpar8(CPAG_04219) cpar9(CPAG_04220) |
| methylglyoxal pathway | cpar3174(CPAG_01315) cpar3176(CPAG_01317) |
| pantothenate and coenzyme A biosynthesis | cpar445(CPAG_03848) cpar446(CPAG_03849) |
| lipid-linked oligosaccharide biosynthesis | cpar3650(CPAG_00799) cpar3657(CPAG_00806) |
| superpathway of glycine biosynthesis ^(sig)^ | cpar800(CPAG_01375) cpar808(CPAG_01383) |
| galactose degradation ^(sig)^ | cpar4835(CPAG_04855) cpar4836(CPAG_04856) cpar4838(CPAG_04858) cpar4839(CPAG_04859) |
| tRNA charging pathway | cpar5036(CPAG_01275) cpar5042(CPAG_01281) ^(2)^  cpar924(CPAG_03455) cpar926(CPAG_03457) cpar927(CPAG_03458) ^(5)^  cpar3228(CPAG_03892) cpar3235(CPAG_03899) ^(6)^ |
| fatty acid oxidation pathway ^(sig)^ | cpar4307(CPAG_02841) cpar492(CPAG_00224) ^(1)^ |
|  | cpar319(CPAG_04190) cpar326(CPAG_04197) ^(2)^ |
| glutathione-glutaredoxin redox reactions ^(sig)^ | cpar3633(CPAG_02350) cpar3634(CPAG_02351) cpar3635(CPAG_02352) cpar3636(CPAG_02353) ^(1)^ |
| Acrylonitrile degradation | cpar950(CPAG_03481) cpar951(CPAG_03482) |

| **Pathway** | **Clustered Genes** |
| --- | --- |
| superpathway of histidine, purine and pyrimidine biosynthesis | LELG_04119 LELG_04127 ^*^ |
|  | LELG_01293 LELG_01302 ^(2)^ |
| aerobic respiration (cyanide sensitive) ^(sig)^ | LELG_00919 LELG_00922 LELG_00930 |
| N-acetylglucosamine degradation ^(sig)^ | LELG_03674 LELG_03675 LELG_03676 |
| methylglyoxal pathway | LELG_01093 LELG_01095 |
| pantothenate and coenzyme A biosynthesis | LELG_04009 LELG_04010 |
| lipid-linked oligosaccharide biosynthesis ^(sig)^ | LELG_04866 LELG_04875 LELG_04879 ^*^ |
| phenylalanine, tyrosine and tryptophan biosynthesis | LELG_05645 LELG_05648 |
| galactose degradation ^(sig)^ | LELG_01645 LELG_01646 LELG_01648 LELG_01649 |
| tRNA charging pathway ^(sig)^ | LELG_00240 LELG_00246 ^(2)^ |
|  | LELG_04026 LELG_04031 ^(4)^ |
|  | LELG_05174 LELG_05175 LELG_05177 ^(5)^ |
|  | LELG_05488 LELG_05496 ^(6)^ |
| glutathione-glutaredoxin redox reactions ^(sig)^ | LELG_02799 LELG_02800 LELG_02801 ^(1)^ |
| Acrylonitrile degradation ^(sig)^ | LELG_05156 LELG_05157 |

| **Pathway** | **Genes Clustered** |
| --- | --- |
| aerobic respiration (cyanide sensitive) ^(sig)^ | DEHA0B04389g DEHA0B04557 DEHA0B04609g |
| N-acetylglucosamine degradation ^(sig)^ | DEHA0F26048g DEHA0F26070g DEHA0F26092g |
| ergosterol biosynthesis | DEHA0F09603g DEHA0F09757g |
| biotin biosynthesis ^(sig)^ | DEHA0G26235g DEHA0G26257g |
| pantothenate and coenzyme A biosynthesis | DEHA0G14179g DEHA0G14190g |
| lipid-linked oligosaccharide biosynthesis | DEHA0C04576g DEHA0C04752g ^*^ |
| arginine degradation (arginase pathway) ^(sig)^ | DEHA0F06556g DEHA0F06754g |
| galactose degradation * ^(sig)^ | DEHA0C02662g DEHA0C02684g |
|  | DEHA0C02838g DEHA0C02860g |
| tRNA charging pathway ^(sig)^ | DEHA0F08228g DEHA0F08404g ^(3)^  DEHA0G21395g DEHA0G21549g ^(4)^  DEHA0C14696g DEHA0C14718g DEHA0C14784g ^(5)^ |
|  | DEHA0F24277g DEHA0F24442g ^(6)^ |
| fatty acid oxidation pathway | DEHA0D18667g DEHA0D18722g ^(1)^ |
| glutathione-glutaredoxin redox reactions ^(sig)^ | DEHA0D17677g DEHA0D17699g ^(1)^ |

| **Pathway** | **Genes Clustered** |
| --- | --- |
| superpathway of histidine, purine and pyrimidine biosynthesis | PICST_34864 PICST_70259 PICST_75662 ^(2)^ |
| aerobic respiration (cyanide sensitive) ^(sig)^ | PICST_35527 PICST_67314 PICST_88762 |
| 2-keto glutarate dehydrogenase complex | PICST_36951 PICST_68297 |
| N-acetylglucosamine degradation ^(sig)^ | PICST_32526 PICST_48829 PICST_62706 |
| tetrapyrrole biosynthesis | PICST_47071 PICST_59732 |
| pantothenate and coenzyme A biosynthesis | PICST_41000 PICST_54699 |
| lipid-linked oligosaccharide biosynthesis ^(sig)^ | PICST_58095 PICST_83561 PICST_89290 ^*^ |
| arginine degradation (arginase pathway) | PICST_35750 PICST_77538 |
| galactose degradation ^(sig)^ | PICST_82625 PICST_76750 PICST_43810 PICST_57493 |
| tRNA charging pathway ^(sig)^ | PICST_77216 PICST_57984 ^(2)^ |
|  | PICST_72398 PICST_77997 ^(3)^ |
|  | PICST_81660 PICST_76328 ^(4)^ |
|  | PICST_33068 PICST_84407 PICST_84563 ^(5)^ |
|  | PICST_72287 PICST_89978 ^(6)^ |
|  | PICST_56543 PICST_88206 ^*^ |
| fatty acid oxidation pathway | PICST_39759 PICST_75424 ^(1)^ |
| glutathione-glutaredoxin redox reactions ^(sig)^ | PICST_35190 PICST_56049 ^(1)^ |
| Acrylonitrile degradation | PICST_33055 PICST_48268 |

| **Pathway** | **Genes in cluster** |
| --- | --- |
| superpathway of histidine, purine and pyrimidine biosynthesis | PGUG_03140 PGUG_03145 ^(1)^ * |
|  | PGUG_02911 PGUG_02918 ^(2)^ |
| aerobic respiration (cyanide sensitive) | PGUG_03697 PGUG_03701 |
| N-acetylglucosamine degradation ^(sig)^ | PGUG_00478 PGUG_00479 PGUG_00480 |
| sphingolipid metabolism | PGUG_01720 PGUG_01730 |
| ergosterol biosynthesis | PGUG_01489 PGUG_01496 |
| pantothenate and coenzyme A biosynthesis | PGUG_04358 PGUG_04359 |
| lipid-linked oligosaccharide biosynthesis | PGUG_05778 PGUG_05788 * |
| arginine degradation (arginase pathway) | PGUG_01375 PGUG_01382 |
| galactose degradation ^(sig)^ | PGUG_05862 PGUG_05863 PGUG_05865 PGUG_05866 |
| tRNA charging pathway ^(sig)^ | PGUG_01430 PGUG_01438 ^(3)^ |
|  | PGUG_03043 PGUG_03047 ^(4)^  PGUG_05629 PGUG_05630 PGUG_05633 ^(5)^  PGUG_00406 PGUG_00414 ^(6)^ |
| fatty acid oxidation pathway | PGUG_00645 GUG_00646 ^(1)^ |
| glutathione-glutaredoxin redox reactions ^(sig)^ | PGUG_00604 PGUG_00605 PGUG_00606 ^(1)^ |

| **Pathway** | **Clustered genes** |
| --- | --- |
| aerobic respiration (cyanide sensitive) | CLUG_03087 CLUG_03097 |
| N-acetylglucosamine degradation ^(sig)^ | CLUG_02904 CLUG_02905 CLUG_02911 |
| ergosterol biosynthesis | CLUG_05112 CLUG_05120 |
| pantothenate and coenzyme A biosynthesis | CLUG_03006 CLUG_03007 |
| starch degradation | CLUG_03470 CLUG_03471 |
| arginine degradation (arginase pathway) ^(sig)^ | CLUG_00267 CLUG_00276 |
| galactose degradation ^(sig)^ | CLUG_02289 CLUG_02291 CLUG_02292 CLUG_02294 |
| tRNA charging pathway | CLUG_01795 CLUG_01799 ^(4)^  CLUG_01664 CLUG_01665 CLUG_01667 ^(5)^ |
| fatty acid oxidation pathway | CLUG_00696 CLUG_00697 ^(1)^ |
| glutathione-glutaredoxin redox reactions ^(sig)^ | CLUG_03150 CLUG_03155 ^(1)^ |
